# Bacterial variability in the mammalian gut captured by a single-cell synthetic oscillator

**DOI:** 10.1038/s41467-019-12638-z

**Published:** 2019-10-11

**Authors:** David T. Riglar, David L. Richmond, Laurent Potvin-Trottier, Andrew A. Verdegaal, Alexander D. Naydich, Somenath Bakshi, Emanuele Leoncini, Lorena G. Lyon, Johan Paulsson, Pamela A. Silver

**Affiliations:** 1 Department of Systems Biology, Harvard Medical School, Boston, MA USA; 2 Wyss Institute for Biologically Inspired Engineering, Harvard University, Boston, MA USA; 3 Image and Data Analysis Core, Harvard Medical School, Boston, MA USA; 4 Harvard John A. Paulson School of Engineering and Applied Sciences, Cambridge, MA USA; 5 Present Address: Department of Infectious Disease, Imperial College London, London, UK; 6 Present Address: Biology Department, Concordia University, Montreal, QC Canada; 7 Present Address: Department of Engineering, Cambridge University, Cambridge, UK

**Keywords:** Synthetic biology, Cell division, Bacterial host response, Oscillators

## Abstract

Synthetic gene oscillators have the potential to control timed functions and periodic gene expression in engineered cells. Such oscillators have been refined in bacteria in vitro, however, these systems have lacked the robustness and precision necessary for applications in complex in vivo environments, such as the mammalian gut. Here, we demonstrate the implementation of a synthetic oscillator capable of keeping robust time in the mouse gut over periods of days. The oscillations provide a marker of bacterial growth at a single-cell level enabling quantification of bacterial dynamics in response to inflammation and underlying variations in the gut microbiota. Our work directly detects increased bacterial growth heterogeneity during disease and differences between spatial niches in the gut, demonstrating the deployment of a precise engineered genetic oscillator in real-life settings.

## Abstract (web summary)

Synthetic gene oscillators can be used to control timed function and periodic expression of genes. Here the authors demonstrate in vivo implementation in the mammalian gut that can keep time over several days.

## Introduction

Oscillators are extensively utilized as clocks and timers in computation and biology^1, 2^. Synthetic gene oscillators in bacterial cells have been developed and refined over past decades^3–12^, led by the development of the repressilator circuit—a simple negative feedback loop constructed from three transcriptional repressor proteins, which inhibit each other’s expression in turn (Tn10 TetR, bacteriophage λ CI and *Escherichia coli* LacI)^3^. Engineered bacteria hold great promise for deployment as diagnostics and therapeutics in the clinic^13^. Oscillatory gene control could enable increasingly complex applications for engineered bacteria, for example controlling time-linked functionality or sophisticated recorders and counters.

Differential bacterial growth underlies colonization of the gut by commensal species during childhood, pathogenic infection, and the establishment of dysbiosis in the microbiota that has been linked to an increasing array of diseases^14^. Recently, several methods have been developed to estimate instantaneous, average bacterial growth rates using metagenomic sequencing data^15–18^. These methods have provided insights into spatial and temporal changes in comparative growth rate^19, 20^. However, single-cell methods offer different advantages, in particular providing valuable information about growth variability across a population^21–29^. Such information is crucial to our understanding of bacterial growth in the gut, which includes a broad range of niches with different growth favorability for any given bacterial species, potentially limiting the interpretation of average growth measures. Similarly, methods that can quantify absolute, rather than relative, growth and that assess growth in aggregate^21, 30^, rather than as instantaneous rates, are particularly important for developing a comprehensive understanding of growth in a non-invasive manner.

**Fig. 1:**
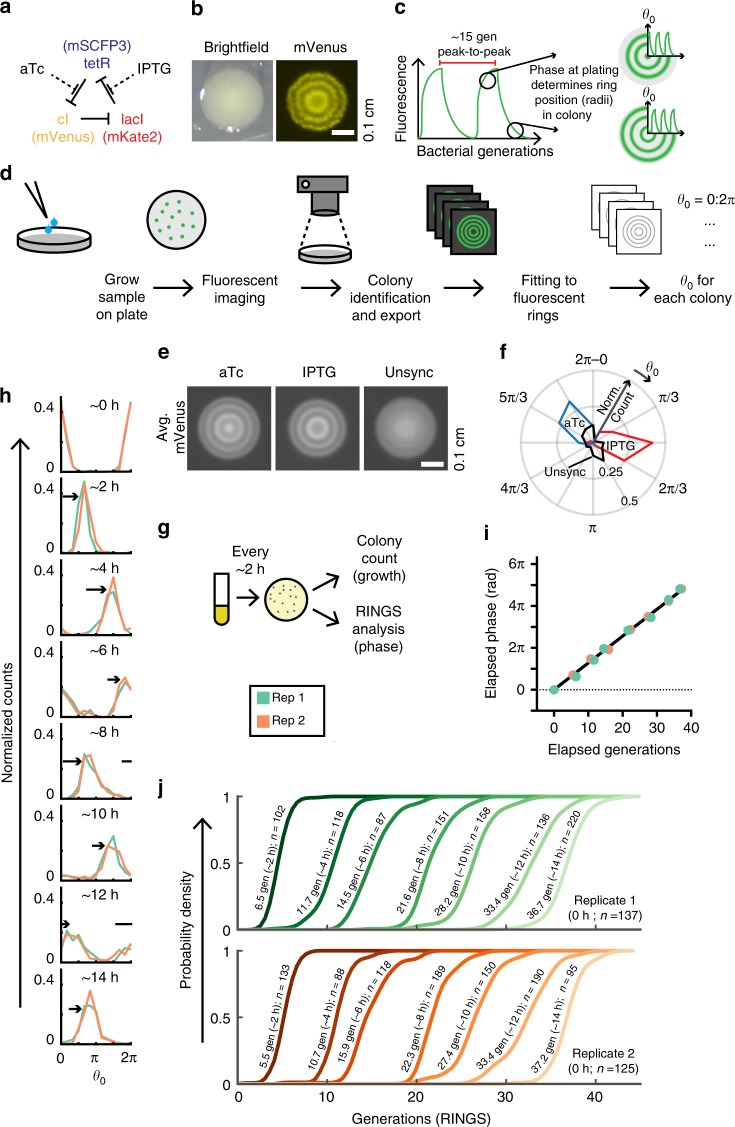
RINGS analysis measures single-cell repressilator 2.0 phase. **a** The repressilator is a ring-oscillator comprising three repressors. **b** Colonies expressing the repressilator 2.0 display fluorescent rings controlled by the circuit’s oscillations. **c** Repressilator phase progresses based on bacterial divisions, with an ~15 generation period. The phase of the colony-initiating bacterium at the time of plating (*θ*_0_) controls the position of the fluorescent rings forming within that colony. **d** The RINGS pipeline consists of plating of a bacterial population on agar plates, followed by imaging, computational identification and export of individual, centered colonies and fitting of a generative model. The fit of ring position is then used to estimate *θ*_0_ at the time of plating. **e** LPT239 bacteria, carrying the repressilator, are synchronized by exposure to aTc or IPTG as demonstrated by average projections of aligned colony images compared to unsynchronized controls. Scale = 0.1 cm. **e** RINGS analysis demonstrates the ability of the repressilator to report on bacterial phase. Graph shows polar histogram representing normalized counts of colonies (radius) within a given *θ*_0_ range (angle). For **e**, **f** IPTG *n* = 93; aTc *n* = 62; unsync *n* = 49. **g** LPT320 bacteria were synchronized with aTc and grown in log-phase growth at 37 °C, plating samples for colony counts and RINGS analysis every 2 h ± 15 min. **h** RINGS analysis measured phase (*θ*_0_) across the population. Graphs show histograms of normalized *θ*_0_ count for two biological replicates. Arrows indicate shift in population since last timepoint **i**. Repressilator phase correlates with the estimated generations as determined by CFU counts from agar plating. Graph shows mean with 95% CI (error bars smaller than datapoints) of elapsed phase after removal of aTc. **j** Cumulative distribution functions of predicted generations elapsed across the population. Labels list average generations for each timepoint as calculated by CFU counts in panel **i**. For **h**–**j**, the number of colonies analyzed are listed in **j**. Source data are provided as a Source Data file

The original repressilator was recently redesigned to afford a highly regular and robust oscillator with reduced error propagation and information losses, referred to herein as the repressilator 2.0 (Fig. 1a; Supplementary Fig. 1)^5^. During in vitro growth the circuit keeps phase in single cells for hundreds of bacterial generations, with an oscillation period that is growth-rate independent and linked to bacterial divisions^5^. The circuit’s low variance between cells, and independence from external feedback and entrainment cues make it an attractive timer for use in complex environments.

Here, we use the repressilator 2.0 to infer bacterial growth dynamics at a single-cell level. We develop and validate an imaging analysis pipeline to determine repressilator phase. In this way, we investigate bacterial population dynamics during colonization of, and growth within, the mammalian gut. Our results show increased growth variability under inflamed conditions, demonstrating the importance of single-cell methods for reliable bacterial growth analysis. We also reveal robust functionality and controllability of the repressilator 2.0 across diverse host and environmental contexts.

## Results

### RINGS method development and testing

Because bacterial colonies expand radially in a uniform manner, with division only occurring at the periphery^31^, synchronous repressilator 2.0 oscillations create stable macroscopic rings when fluorescent reporters are driven under repressilator 2.0 control (Fig. 1b)^5^. The phase of a bacterium that seeds a colony determines the phase, and thus fluorescent reporter expression, of each subsequent generation throughout the colony. As such, the positions (i.e., radii) of the fluorescent rings in a colony are indicative of the phase of the bacterium that seeded the colony (Fig. 1c).

To utilize this behavior, we developed a workflow for bacterial colony image capture and processing, which we call Repressilator-based INference of Growth at Single-cell level (RINGS) (Fig. 1d). The process begins with fluorescent macroscope imaging of repressilator 2.0-expressing bacterial colonies. Image analysis then identifies colonies and fits a generative model to the fluorescent rings within each colony. The ring positions are used to estimate the relative phase of the single bacterium that seeded the colony at the time of plating (*θ*_0_). Because repressilator 2.0 progression is linked to bacterial growth^5^, the elapsed phase between two timepoints can be used to infer bacterial growth. Given the periodic nature of the oscillator, meaning that the circuit’s output is the same in any given period (i.e., values are modulo 2π), adjusting the sampling frequency to ensure that less than a period elapses between timepoints allows unambiguous determination of growth.

*E.coli* LPT239 bacteria expressing the repressilator 2.0 (Supplementary Table 1) were phase-synchronized by growth in the presence of Isopropyl β-D-1-thiogalactopyranoside (IPTG) or anhydrotetracycline (aTc), which interrupt repression by LacI and TetR, respectively (Fig. 1a). Analysis of YFP fluorescence by colony imaging (Fig. 1e) and RINGS analysis (Fig. 1f) demonstrated the ability for RINGS to distinguish between these two distinct oscillator phases. Further optimization through variation of the ‘sponge plasmid’ (Supplementary Fig. 1), a key element in previous efforts to reduce oscillation variability in the repressilator 2.0 circuit^5^, yielded more consistent fluorescent rings within colonies and allowed subsequent RINGS analyses using combined CFP and YFP fluorescence data (Supplementary Fig. 2).

To further test the ability of RINGS to track bacterial growth over time, we sampled aTc-synchronized *E. coli* LPT320 bacteria (Supplementary Table 1), kept in constant log-phase culture by back-dilution and plated at ~2-h intervals. Plates were imaged and analyzed using RINGS to measure repressilator phase, and for colony forming unit (CFU) counts to measure average bacterial growth (Fig. 1g). Repressilator 2.0 phase progressed coherently according to bacterial growth of the population (Fig. 1h, i, Supplementary Fig. 3a, b), with regression of the elapsed phase (RINGS) vs growth (CFU) data quantifying the LPT320 repressilator 2.0 period length as 15.4 ± 0.2 SEM gen/period (Fig. 1i). This is consistent with previous period measures of similar circuits^5^. The period length allows assignment of a distribution of probable growth on a single-cell level, forming a cumulative probability distribution for the population (Fig. 1j).

### RINGS is robust to strain and environmental variations

**Fig. 2:**
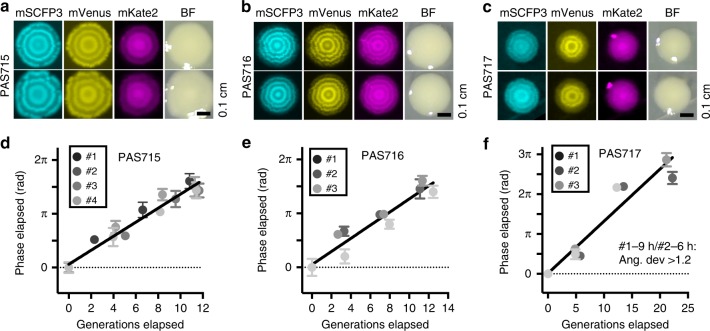
RINGS can assess growth in different bacterial strains and species. **a**–**c** Fluorescence and white light images of two representative colonies of PAS715 (*E. coli* MG1655), PAS716 (*S*. Typhimurium LT2), and PAS717 (*E. coli* Nissle 1917). Scale bars = 0.1 cm. **d**–**f** When grown in log-phase liquid culture, repressilator phase progression correlated with the estimated generations elapsed as determined by CFU counts for PAS715 (four biological replicates), PAS716 (three biological replicates), and PAS717 (three biological replicates). Graphs also show linear regression from combined means of elapsed phase after IPTG removal. Error bars = 95% CI. Numbers are as follows: PAS715: replicate #1/2/3/4: 0 h:16/76/23/89 2 h:8/14/42/40 4 h:25/15/10/12 6 h:9/11/40/46. PAS716: replicate #1/2/3 – 0 h:145/33/22 2 h:76/43/19 4 h:94/106/18 6 h:32/19/18. PAS717: replicate #1/2/3. 0 h: 116/37/69 3 h: 48/60/96 6 h: 78/41/76 9 h: 48/42 /67. #1–9 h and #2–6 h data had angular deviation >1.2 and were removed from downstream analysis. Source data are provided as a Source Data file

The repressilator was transferred to *E. coli* (MG1655 - PAS715; and the human probiotic strain, Nissle 1917 – PAS717) and *Salmonella enterica* serovar Typhimurium (hereafter referred to as *S*. Typhimurium) (LT2-PAS716) strains (Fig. 2a–c; Supplementary Table 1), where RINGS again faithfully tracked cell divisions (Fig. 2d–f). The period of the repressilator was determined in each case either using the regression of RINGS vs growth curves (Fig. 2d–f) or during growth in a microfluidic device capable of trapping cells for fluorescent microscopic analysis over multiple repressilator cycles (Supplementary Fig. 4a, b; Table 1)^32^. Both methods quantified the same period for *E. coli* PAS715 bacteria grown in rich medium (RINGS: 15.3 ± 0.8 SEM gen/period; mother machine: 15.3 ± 0.2 SEM gen/period) (Fig. 2d; Table 1), with a similar period also calculated for *S*. Typhimurium PAS716 and *E. coli* Nissle 1917 PAS717 bacteria using RINGS (PAS716: 16.4 ± 1.5 SEM gen/period; PAS717: 15.3 ± 1.5 SEM gen/period) (Fig. 2e, f).

**Table 1:** Repressilator period lengths calculated by growth in the mother machine

Strain	Conditions	Calculated period (gen/period ± SEM)	*n*
PAS715	Rich medium	15.3 ± 0.2	202
PAS715	Cecum contents medium	15.6 ± 0.4	123
PAS718^a^	Cecum contents medium (fresh)	15.5 ± 0.1	552
PAS718^a^	Cecum contents medium, untreated mice (frozen)	15.6 ± 0.1	353
PAS718^a^	Cecum contents medium, DSS-treated mice (frozen)	15.6 ± 0.2	186

^a^PAS718 = PAS715 expressing a constitutive fluorescent marker for accurate cell segmentation

**Fig. 3:**
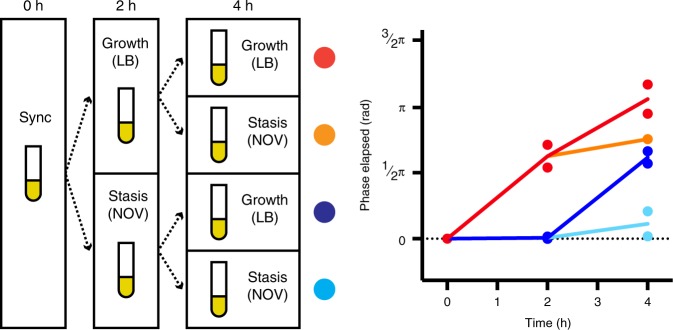
RINGS is unaffected by variable bacterial growth. To test the ability for RINGS analysis to track and integrate growth under variable conditions, IPTG-synchronized PAS715 was back-diluted and grown in liquid culture in the presence or absence of bacteriostatic concentrations of novobiocin. Following 2 h of growth, each culture was washed and split again to be grown under the presence or absence of novobiocin, generating four unique combinations of expected growth and non-growth. The phase of populations sampled at 2 and 4 h were consistent with the expected growth phenotype. In particular, the equivalence of the two populations exposed to one period with and one period without novobiocin, irrespective of order, demonstrates the ability to measure absolute growth during periods of variable growth rate. Mean phase (*θ*_0_) from two independent biological replicates is shown. Numbers of colonies analyzed per timepoint are as follows: Rep 1/2 – 0 h: 13/20. 2 h LB: 57/26. 2 h novo: 101/69. 4 h LB-LB: 14/60. 4 h LB-novo: 23/42. 4 h novo-LB: 40/32. 4 h novo-novo: 22/16. Source data are provided as a Source Data file

PAS715 period length remained unperturbed during growth on extracted mouse cecum contents, used to simulate aspects of the gut environment (Supplementary Fig. 4a; Table 1). Similarly, expected growth variations caused by alternating the presence or absence of the bacteriostatic antibiotic novobiocin were accurately tracked using RINGS analysis (Fig. 3). Together, these results indicate that the mechanisms for repressilator 2.0 oscillation are insensitive to the gene regulation, cell-size, and stress variations under changing species backgrounds and environmental conditions. This provides confidence both for the robustness of the RINGS method, and the possibility of extending its use to other applications and engineerable gut bacterial strains.

### RINGS quantifies growth changes within the mouse gut

**Fig. 4:**
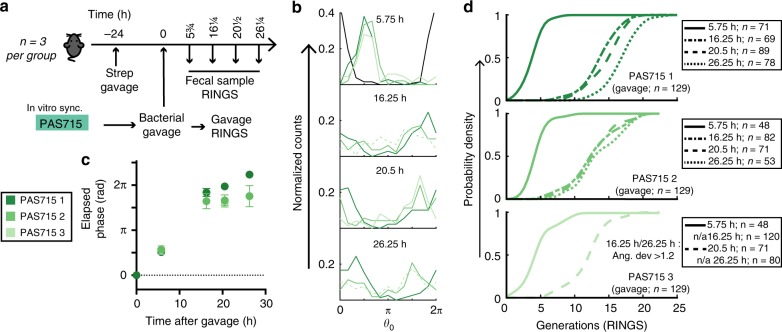
The repressilator 2.0 is robust and informative within the mouse gut. **a** in vitro synchronized PAS715 (*E. coli*) bacteria measure growth in mice treated with a single dose of streptomycin (*n* = 3 per group). **b** Histograms of bacterial phase distributions throughout the experiment. Graphs show normalized counts. 5.75 h timepoint is compared to the gavage population (black line). Dotted lines represent datasets with angular deviation >1.2, which were excluded from downstream analyses. For number of colonies analyzed at each timepoint, see corresponding data in **d**. **c** Graph of average elapsed phase of the population vs time for each mouse. Graph shows mean with 95% CI. **d** Cumulative distribution functions of predicted generations elapsed since gavage. Source data are provided as a Source Data file

IPTG-synchronized *E. coli* PAS715 bacteria were delivered by oral gavage to mice that had been treated with a single dose of streptomycin 24-h prior (Fig. 4a; replicate: Supplementary Fig. 5a). Streptomycin-treated mice have been extensively utilized as a model for *E. coli* and *S*. Typhimurium growth in the mouse gut, which is not a native or permissive host for human-derived *E. coli* strains^33, 34^. We have previously shown that delivery of *E. coli* to mice that were treated with single doses of streptomycin affords colonization levels that are initially high but decrease consistently over the days following^35, 36^. This likely reflects rapid initial replication in the niche made available by streptomycin treatment, followed by progressive recovery of colonization resistance by the native gut and microbiota^37^. Thus, this model allows us to investigate the ability for RINGS to detect a range of growth rates within the gut. RINGS analysis was performed on *E. coli* PAS715 bacteria isolated from fecal samples (Fig. 4a–d; replicate: Supplementary Fig. 5a–e). Repressilator phase remained remarkably coherent in populations for >20 h after gavage (Fig. 4b; replicate: Supplementary Fig. 5b, c). Similar results were found for *S*. Typhimurium LT2 (PAS716) (Supplementary Fig. 5g–j).

The growth of each species was quantified using the previously calculated repressilator 2.0 periods (Fig. 2d–f; Table 1), with physiological constraints for bacterial growth used to assist in the confident assignment of elapsed growth from RINGS phase data (Supplementary Fig. 6). As expected, RINGS analysis estimated that growth was most rapid immediately following gavage and slowed considerably in later timepoints (Fig. 4c, Supplementary Fig. 5d, i), with similar average growth predicted for both species (PAS715: 12.2–15.1 gen and PAS716: 13.0–15.1 gen at 20.5 h post gavage). These dynamics were further confirmed by Peak-to-Trough Ratio (PTR) analysis, which estimates the average instantaneous growth rate of a population from metagenomic sequencing data (6 h: 1.72 ± 0.08, *n* = 4; >16 h: 1.44 ± 0.08, *n* = 9; Supplementary Table 2)^15^. Together these data demonstrate the ability for RINGS to detect changing growth within the mouse gut.

### In vivo repressilator 2.0 control and stability

**Fig. 5:**
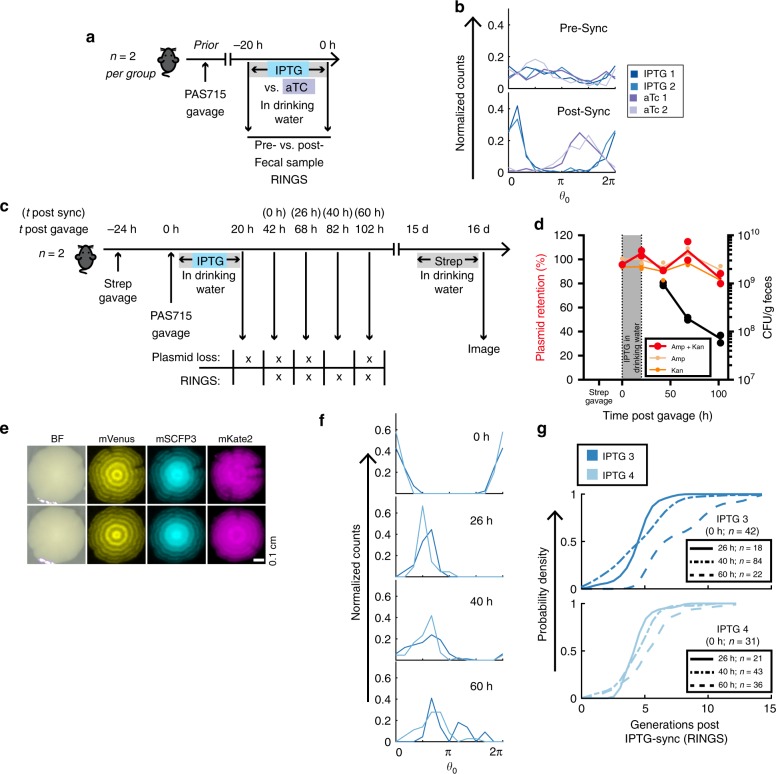
The repressilator 2.0 can be controlled in situ within the mouse gut. **a** Mice (*n* = 2 per group) carrying asynchronous PAS715 were provided IPTG or aTc in drinking water to synchronize the repressilator. **b** RINGS analysis demonstrated the progression from pre- (top) to post-synchronization (bottom). Graph shows histograms of normalized phase count distributions. Numbers are as follows: IPTG1/2—pre: 133/97. Post: 172/206. aTc1/2—pre:149/114. post: 213/193. **c**. Repressilator in situ synchronization allows RINGS analysis following initial colonization, with bacteria plated on selective plates to assay for repressilator and sponge plasmid retention, or RINGS analysis to follow repressilator phase. After 15 days, provision of streptomycin in drinking water overnight selected remaining PAS715 bacteria to allow isolation and imaging of colonies that had been continually present in the gut. **d** Plasmid retention (colored samples, left axis) and bacterial abundance (black samples, right axis) data from plating on differentially selective plates. Graph shows individual values and mean line. Numbers are as follows: rep1/2 – 20 h: 772/443. 42 h: 1041/905. 68 h: 1967/1742.102 h: 585/841. **e** Fluorescence and white light images of two representative colonies of PAS715 isolated 16 days post gavage. Scale bars = 0.1 cm. **f** RINGS analysis of phase progression 42 h (0 h post sync) -102h (60 h post sync) after gavage. Graphs show histograms of normalized bacterial phase count distributions. For number of colonies analyzed at each timepoint, see corresponding data in **g**. **g **Cumulative distribution functions of predicted generations elapsed since gavage. Source data are provided as a Source Data file

To allow in vivo control of the repressilator 2.0 circuit and re-gain synchronicity of the bacterial populations growing within the mouse gut, mice carrying asynchronous PAS715 bacteria were provided IPTG or aTc in their drinking water (Fig. 5a). RINGS analysis on fecal samples in pre- and post-synchronized mice clearly demonstrated the ability to synchronize the repressilator within the mouse gut (Fig. 5b).

The repressilator circuit was stable over extended periods of growth in the gut. To test plasmid retention, *E. coli* PAS715 bacteria were provided to mice previously treated with a single dose of streptomycin. Plating of fecal samples was then used to identify plasmid loss events within the bacteria (Fig. 5c). Retention of both the repressilator 2.0 plasmids remained high for >100 h in the mouse gut (80–88% retention at 102 h post gavage), indicating that the repressilator circuit can remain active over these time frames (Fig. 5d). Furthermore, functional PAS715 colonies could still be isolated 16 days after first entering the gut (Fig. 5e), demonstrating the potential for this synthetic circuit to maintain longer-term oscillatory gene expression in a competitive environment.

### Long-term in vivo growth determination using RINGS

Repressilator 2.0 circuit stability and in situ synchronization capacity allows for long-term growth determination. To measure growth after initial establishment in the gut, during plasmid retention testing PAS715 bacteria were synchronized in situ with IPTG (Fig. 5c). Based on our findings that bacterial growth slowed over the initial 24-h period (Fig. 4a–d, Supplementary Fig. 5a–e) RINGS analysis was performed beginning the day after IPTG removal on fecal samples taken ~daily, with well under a single period of growth expected between each timepoint (Fig. 5f). Repressilator phase remained coherent (Fig. 5f) predicting 6–12 generations (IPTG 3, 90% CI) and 5–9 generations (IPTG 4, 90% CI) of growth over the 60-h period beginning ~2 days after gavage (Fig. 5g).

### RINGS detects disease and niche-specific growth variations

**Fig. 6:**
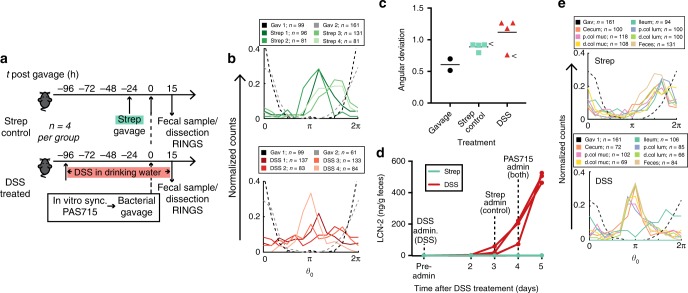
RINGS identifies increased growth variability in the inflamed gut and between gut niches. **a** RINGS was used to compare PAS715 growth between the dextran sulfate sodium (DSS) inflamed, or streptomycin treated, mouse gut. **b** Histograms of normalized bacterial phase counts in gavage (black and grey) and fecal samples extracted 15 h post gavage in streptomycin (green) and DSS (red) treated mice **c** Bacteria extracted from feces of DSS-treated mice (red) showed elevated RINGS variability compared to gavage (black) and streptomycin (green) treated mice. Graphs show angular deviation of each datapoint and mean. Arrows identify mice dissected and analyzed in **e**. **d** Quantification of LCN-2 in feces 15 h post gavage demonstrates elevated inflammation in DSS-treated mice. **e** Bacteria plated from the dissected mouse gut of streptomycin control (top; *n* = 1) and DSS-treated (bottom; *n* = 1) mice. Graphs show histograms of normalized bacterial phase counts from populations dissected from regions of the gut (various colors) and feces (brown) compared to the original gavage (black). Source data are provided as a Source Data file

Different growth behavior was determined in the inflamed gut and between regions of the gut. Repressilator-bearing PAS715 bacteria were introduced into dextran sulfate sodium (DSS) -treated animals as a model for inflammation, which promotes increased growth of *Enterobacteriaciae*^38, 39^ (Fig. 6a). Bacterial populations retrieved from the feces of DSS-treated mice 15 h after delivery showed greater phase heterogeneity than those from streptomycin-treated control mice (Fig. 6b), as demonstrated by comparison of the angular deviation, the circular statistical analogue to standard deviation, of each population (strep control: 0.9 ± 0.0 SEM, *n* = 4; DSS-treated: 1.1 ± 0.1 SEM, *n* = 4) (Fig. 6c). These results were consistent with previous experiments that showed signs of decoherence of PAS715 populations <20 h in the inflamed gut (Supplementary Fig. 7a, b). DSS (4%) did not affect the repressilator during in vitro growth (Supplementary Fig. 7c) and repressilator 2.0 period length was not affected by growth on cecum contents extracted from either untreated or DSS-fed mice (Table 1). Together these results suggest that the DSS-inflamed gut provides a more heterogeneous growth environment than the streptomycin-treated gut.

Interestingly, despite all DSS-treated mice showing equivalent severity of inflammation, as measured by lipocalin 2 (LCN-2) levels (Fig. 6d), PAS715 remained relatively synchronous in one of the DSS-treated mice, with an angular deviation of the fecal population similar to that seen in streptomycin-treated mice (Fig. 4c, arrow). To further analyze bacterial growth within this mouse, we dissected the lower gastrointestinal tract and separately plated bacteria extracted from distinct regions of the gut: ileum, cecum, proximal colon lumen, proximal colon mucus, distal colon lumen, distal colon mucus, and feces (Fig. 6e). Dissection analysis of a streptomycin control mouse showed similar RINGS phase distributions for all regions measured (Fig. 6e, top). By comparison, populations extracted from the DSS-treated mouse showed similar RINGS phase distributions in all regions except the ileum, which was distinctly phase shifted (Fig. 6e, bottom). Thus, even when not reflected in feces, greater PAS715 growth heterogeneity was evident in the DSS-treated compared to streptomycin-treated gut. The difference in growth elapsed between bacteria in the ileum and the remainder of the DSS-treated gut is a likely cause for the increased growth variability we detect in fecal samples from other DSS-treated mice.

## Discussion

Here we describe the activity of a synthetic gene oscillator, the repressilator 2.0, in the complex environs of the mammalian gut. We develop an image processing and analysis pipeline, RINGS, to follow repressilator activity on a single-cell level and thereby also understand growth dynamics at various stages of the colonization process. We demonstrate robust repressilator 2.0 functionality, control and circuit stability within the mouse gut over the course of several days. Using this method we detect increased growth variability under inflammatory conditions and between spatial niches within the gut

The repressilator 2.0 is ideally suited to periodic expression control within engineered bacteria for use in complex settings. The circuit’s independence from external signals allows for circuit function in a variety of environments, which is of particular utility in the gut where relative abundance of strains can vary widely on spatial and temporal scales, and between individuals. To this end, the ability to re-synchronize the repressilator 2.0 in situ within the gut, and its stability over extended periods of growth without antibiotic selection are particularly exciting.

The RINGS method informs us of the integrated growth experience of a bacterium and its forebears. The repressilator 2.0’s periodicity allows for extend growth measurement in comparison to other comparable techniques, recent examples of which include a synthetic particle which allows mark-and-recapture analysis over ~14 generations^30^, and fluorescent protein dilution which is capable of estimating up to ~10 divisions of single-cell growth^21^. Our analyses estimated *E. coli* MG1655 division times progressively increasing from ~35 min, when colonizing the recently streptomycin-treated gut, to ~20 h when more removed from the perturbation of the single dose of streptomycin and thus reflecting more physiological growth conditions. By comparison, a recent study used the accumulation of mutations in the *E. coli* genome to estimate a division time in humans of 15 h^40^.

Together, our results suggest that the aggregate bacterial experience is relatively consistent under most scenarios tested, with spatial dissection suggesting that the majority of growth in these mouse colonization models is occurring ‘upstream’ in the small intestine and/or cecum. We see that in most cases fecal sampling accurately reflected the overall growth profile of the mouse colon, including bacteria in the lumen and mucus layers, and within the cecum. The absence of colonies in fecal samples reflecting the differential growth observed in the ileum of the dissected DSS-treated mouse suggests that, in this case at least, the bacteria from the ileum either were not represented in feces or represent a small enough percentage of the population so as to remain below detection in our final analyses from this mouse.

Overall, we determine that single-cell measurements are particularly important in disease conditions, such as inflammation, when variability of growth between niches is accentuated. In these cases methods that can only measure a population-wide average would not adequately represent the population’s diversity. The ability to increase the sampling frequency used would also allow this method to measure the emergence of subpopulations of differentially growing bacteria in finer detail.

In sum, analysis of the repressilator at single-cell level can report on complex bacterial growth behaviors over time, and in particular, during disease conditions within the mammalian gut. In doing so, this work demonstrates the potential for one of the circuits that first stimulated the field of synthetic biology to revolutionize how we control gene expression within the gut.

## Methods

### Strains, plasmids, and bacterial culturing

Details of strains (Supplementary Table 1) and plasmids (Supplementary Table 3) used in this study are provided.

The repressilator 2.0 plasmid variant with three fluorescent reporters (pLPT234) was constructed by isothermal assembly^41^ by combining PCR products from previously published degradation-tag free repressor genes—Tn10 transposon derived *tetR*, bacteriophage λ derived *cI* and *E. coli* lactose operon derived *lacI* (pLPT119)—and triple fluorescent reporters—P_R_-mKate2 (including the first 11 amino acids of mCherry for improved translation efficiency as previously published^5^), P_LtetO1_–mVenus, P_LlacO1_–mSCFP3 (pLPT107)^5^. Previously published sponge plasmid variants^5^ were used as available from Addgene plasmid repository (see Data Availability Statement). Plasmids were isolated by miniprep (Qiagen) and were routinely transferred to new strains as a mixture by electroporation.

To avoid interruption of the repressilator and ensure clear ring development within colonies, *lacI* and *motA* genes were knocked out of *E. coli* strains used before repressilator plasmids were transferred. For both genes, FRT-flanked gene disruption constructs were transferred by P1*vir* transduction^42^ from the relevant Keio collection strains^43^. Kanamycin resistance genes were then removed by electroporation and subsequent curing at 43 °C of pCP20^44^. Similarly, streptomycin resistance based on a *rpsL* lys42arg mutation was transferred by P1*vir* from a previously generated *E. coli* MG1655 strain^35^. Resistance to streptomycin was evolved in *S*. Typhimurium LT2 by serial passage in liquid culture with increasing concentrations of streptomycin sulfate (Sigma) (50,100, 200, 300 μg/mL). For PAS718, mKate2 (including the first 11 amino acids of mCherry for improved translation efficiency as previously published^5^) driven under the P_RNA1_ constitutive promoter was inserted into the genome using a Tn7 transposon^45^, and acted as a constitutive fluorescent marker for cell segmentation in mother-machine experiments.

Bacteria were routinely cultured in Luria broth (LB) supplemented with 300 μg/mL streptomycin (Sigma), 100μg/mL carbenicillin or ampicillin (Sigma) and 50μg/mL kanamycin (Sigma). For plating, bacteria were grown on selective LB agar plates supplemented with 100μg/mL carbenicillin (Sigma) and 50μg/mL kanamycin (Sigma). The plasmid retention test compared growth on streptomycin (all PAS715 derivatives) to streptomycin+carbenicillin (retention of repressilator plasmid), streptomycin+kanamycin (retention of sponge plasmid), and streptomycin+carbenicillin+kanamycin (retention of both plasmids) at drug concentrations as above.

To synchronize the phase of the repressilator across the population, bacteria were grown overnight in the presence of 1 mM Isopropyl β-D-1-thiogalactopyranoside (IPTG) (Sigma) or 100 nm anhydrotetracycline (aTc) (Sigma). Bacteria were back-diluted in fresh inducer-supplemented media by at least 1:20 to allow resumption of active growth in the presence of inducer, before being washed and utilized for downstream experiments.

### Colony imaging

Fluorescent and white light images of colonies were imaged using a custom-software controlled Canon T3i digital single lens reflex (DSLR) camera with a Canon EF-S 60 mm USM lens, combined with LEDs and filters for excitation and a Starlight express emission filter wheel (CFP: 440–460 nm LED with 436/20 EX and 480/40 EM filters; YFP: 490–515 nm LED with 500/20 EX and 530/20 EM filter; RFP: 588–592 LED with 572/35 EX and 645/75 EM filter; white: 3500–4500 K LED)^46^. Images were taken at an aperture of f/2.8 and ISO200. Exposure times were typically between 0.05 and 2 s as experimentally determined to maximize dynamic range.

### Generative model development for RINGS phase profiling

We extract the relative phase offset of each colony by fitting a generative model to the pattern of oscillations. This approach has the benefit of making use of all the information captured in the multi-channel images. We explored a family of generative models, based on the simple form:

1 $${\it{I}} = {\it{A{\mathrm{sin}}}}\left( {f\left( r \right)} \right) + {\it{B}}$$

where *A* and *B* are the amplitude and offset of a sinusoidal oscillator, and the function, *f*(*r*), represents the radial phase profile of the colony. The form of *f*(*r*) is derived below using a simple model for the growth of the colonies.

The phase profile, *f*(*r*), is related to the profile of generation number, *g*(*r*), as follows:

2 $${\it{f}}\left( r \right) = \frac{{2\pi }}{{\it{T}}}{\it{g}}\left( {\it{r}} \right) + \theta _0$$

where *T* is the period of the oscillator in generations (≈ 15.5) and *θ*_0_ is the instantaneous phase of the oscillator at the time of plating. Thus, we derive the profile of generation number with respect to radius. We assume that growth is initially exponential, and then becomes restricted to an annulus with thickness *D* (≈30 μm at the edge of the colony^40^).

Assuming uniform packing of bacteria in the colony (for both phases of growth), the generation number profile during exponential growth can be derived simply:

3 $${\it{g}}\left( r \right) = \frac{{{\mathrm{log}}\left( {\frac{\pi }{{A_{\mathrm{b}}}}r^2} \right)}}{{{\mathrm{log}}\left( 2 \right)}}$$

where *A*_b_ is the area of a single bacterium

Here we model the growth of the colony with each successive generation. With each generation the radius of the colony increases as follows:

4 $$\frac{{{\mathrm{\Delta }}r}}{{{\mathrm{\Delta }}g}} = {\it{r}}\left( {\sqrt {1 + 2\frac{D}{r} - \left( {\frac{D}{r}} \right)^2 - 1} } \right)$$

Integrating this over the colony, we obtain the following expression for the generation number profile:

5 $$\begin{array}{l}{\it{g}}\left( r \right) = \frac{1}{{4D}}\left[ {2r\left( {1 + a\left( r \right)} \right) + 2D{\mathrm{log}}\left( {2r - D} \right) + 3D{\mathrm{log}}\left( {D + r\left( {1 + a\left( r \right)} \right)} \right)} \right.\\ \left. {\quad \quad \quad - D{\mathrm{log}}\left( { - D + r\left( {3 + a\left( r \right)} \right)} \right)} \right] + g_0\end{array}$$

*g*_o_ is the generation number at the transition between the exponential and annular growth.

Combining the two growth models yields a continuous growth curve (Supplementary Fig. 8). We set the transition between the exponential and annular growth at *r* = *D*, with *D* = 30 μm. Notice that beyond *r* ≈ 100 μm the phase profile is approximately linear, and can be described by:

6 $${\it{f}}\left( r \right) = \left( {\frac{{2\pi }}{{DT}}} \right)r + \theta _0$$

We checked this result by measuring the slope for the phase profile directly from images of colonies. The distance between two peaks in any given colony is ~490 μm, yielding a phase profile with slope 0.0128 rad/μm.

Using an approximate value of 15.5 generations per cycle, Eq. 6 predicts an annulus size, *D* ≈ 32 μm, consistent with the ≈30 μm growth annulus reported in previous studies^47^.

Thus, we model the phase profile of a bacterial colony using Eq. 1 with a linear phase profile, described by Eq. 6.

### Image preprocessing

Centered colonies were cropped from whole-plate images using FIJI^48^. Color images from single fluorescent exposures in CFP and YFP channels were imported, and the relevant spectral component image utilized for downstream processing (CFP—blue channel, YFP—Green channel). Colonies were identified by autothresholding (Yen method) of binarized CFP images. Separate YFP and CFP images were cropped and exported, centered on the YFP center of mass of each identified colony. Prior to final analyses, colony images from plates containing >130 bacterial colonies were excluded based on overcrowding leading to limited colony growth. Remaining colonies were visually inspected, and any doublet or severely malformed colonies were removed from the datasets to avoid spurious fitting. Colony removal was performed blinded to *θ*_0_ fit values. Where presented, average projections were taken through YFP images from all colonies identified in a given population.

### RINGS generative model fitting

Preprocessed colony images were normalized for global changes in intensity, before fitting the model described above. We observed that in each colony, the expression of YFP and CFP decayed dramatically with increasing radius. To compensate for this decay, the images were masked at radius of *r*_max_ = 1.3 mm (*r*_max_ = 1.1 mm for *S*. Typhimurium colonies, which were commonly smaller) and the radial intensity profile was fit to a second order polynomial. This smooth polynomial decay, chosen as an even order with negative coefficient to ensure this process did not remove the oscillatory pattern, was then used to normalize the raw image in a pixel-wise fashion (Supplementary Fig. 9). We also explored normalization with a fourth order polynomial, but this was deemed less appropriate. For multi-channel images, the polynomial functions shared parameters for the center of the colony, but each channel was fit to its own decay profile.

To fit the oscillatory pattern of a single colony, we use Eq. 1 with a linear phase profile. However, we allow for both the slope and offset of the phase profile to be fit (Eq. 6), as well as the amplitude and offset of the intensity (Eq. 1). The slope for the phase profile is initialized to an expected value that varies based on strain, and the phase offset is initialized to zero. We tested optionally masking the central region of the colony, to account for the early exponential growth phase, which we do not attempt to model, however, found no benefit to fit based on this. Fitting is done in Matlab using the Levenberg-Marquardt (LM) algorithm (Supplementary Fig. 9).

The fitting routine returns the center position of the colony, the amplitude and offset of the oscillations, as well as the slope and offset of the phase profile. The phase offset is the property of interest. However, fitting the radial expression profile is challenging due to numerous local minima in the error surface. Thus, we filter out colonies for which the slope of the phase profile, or the inferred center position of the colony is outside of an accepted range. We also explored the use of robust error functions, to minimize the effect of asymmetric bright patches of expression in the colony, as well as weighted nonlinear regression to compensate for the increasing number of pixels at increasing radius. However, neither of these approaches greatly improved upon the LM algorithm, which we used by default. We also explored fitting phase profiles with higher order polynomials; however, we found this to be very unstable.

Fitting was improved by simultaneous regression in multi-channel images.

In this case, the phase offset of the two channels was fixed to a constant value. We found that YFP oscillations lead CFP by a strain-specific value. Thus, we regressed the slope and offset of the phase profile using both channels, with a fixed phase difference between the two channels, as calculated for each strain. See Eq. 7 and 8.

7 $${\it{I}}_{{\mathrm{YFP}}} = {\it{A}}_{{\mathrm{YFP}}}{\mathrm{sin}}\left( {kr + \theta _0 + {\mathrm{strain}}\,{\mathrm{offset}}} \right) + B_{{\mathrm{YFP}}}$$

8 $$I_{{\mathrm{CFP}}} = A_{{\mathrm{CFP}}}\sin \left( {kr + \theta _0} \right) + B_{{\mathrm{CFP}}}$$

### RINGS method parameter testing

We tested the performance of this method by applying it to in vitro timecourse data where colony counts could be used to provide a known average generation shift between each timepoint. Parameter sweeps were performed on key parameters expected to vary between strains, particularly the size of colony mask (‘rMax’), the distance between each ring and therefore expected slope of the phase profile (‘expectedSlope’), the variation in slope between colonies (‘slopeTol’), and the offset between CFP and YFP rings (‘colorPhaseShift’). The method was found to be largely insensitive to specific parameter values with strain-specific optimal values likely caused by differences in oscillator and spatial colony growth characteristics. Optimal parameters for each strain in the study were determined to be as follows: (LPT320: ‘rMax’:1.3, ‘expectedSlope’: 0.39, ‘slopeTol’: 0.3, ‘colorPhaseShift’: 1.5. PAS715: ‘rMax’:1.3, ‘expectedSlope’: 0.34, ‘slopeTol’: 0.4, ‘colorPhaseShift’: 0.9. PAS716: ‘rMax’: 1.1, ‘expectedSlope’: 0.43, ‘slopeTol’: 0.4, ‘colorPhaseShift’: 1.0. PAS717: ‘rMax’:1.3, ‘expectedSlope’: 0.41, ‘slopeTol’: 0.3, ‘colorPhaseShift’: 0.83.).

### Statistical analyses

Statistical analyses were performed in MATLAB versions R2017a-2018a (Mathworks) or Prism v6-7 (Graphpad). Circular statistical tests, specifically circular mean and angular deviation, were performed using the CircStat for Matlab toolbox v2012a^49^.

Briefly, to calculate circular mean, *θ*_0_ values are transformed to a resultant mean vector (Eq. 9).

9 $${\mathbf{r}} = {\mathrm{sum}}\left( {\exp \left( {i\theta _0} \right)} \right)$$

The circular mean, **α**, is then calculated from this vector (Eq. 10).

10 $${\mathbf{\alpha }} = {\mathrm{angle}}\left( {\mathbf{r}} \right)$$

Angular deviation, *s*, is a circular statistical analogue of linear standard deviation. It is derived from the length of the resultant mean vector (Eqs. 11 and 12). Angular deviation values are bounded, lying between 0 and √2. Conceptually, this occurs because values equally spread around the unit circle are maximally spread.

11 $${\it{R}} = \left\| {\mathbf{r}} \right\|$$

12 $${\it{s}} = \sqrt {2\left( {1 - {\it{R}}} \right)}$$

### Elapsed phase measurements

Downstream processing of phase measurements was predominantly undertaken using custom scripts in Matlab versions 2016a–2018a. In order to estimate the total phase elapsed during an experiment, we made the assumption that repressilator phases across the population fell within ±π of the circular mean (i.e., we assumed that all colonies lie within ~15 generations elapsed of each other). We deemed this appropriate where the population remained relatively coherent around the circular mean—which we defined as having an angular deviation <1.2. Empirically, datasets with angular distribution >1.2 were routinely decoherent, were not accurately described by the circular mean (e.g., in cases of a bifurcated population), or contained considerable fractions of the population in regions neighboring the mean ± π divide, which reduced our confidence of accurately classifying these cells as being slower or faster growing (Supplementary Fig. 10). While in many cases valuable insight may still be gained by further analysis on a case-by-case basis, particularly when taken in the context of other datapoints or experimental replicates, we conservatively elected to restrict our interpretations of such datasets to the raw *θ*_0_ data, which are unaffected by this.

For elapsed phase calculations, individual phase values (*θ*_0_) were normalized to the circular mean of the initial synchronized repressilator 2.0 population for that experiment, **α**_sync_. Elapsed phase was then calculated by addition/subtraction of multiples of 2π based upon the circular mean of a timepoint. When **α**_n_ > **α**_**n-1**_ i.e., when growth had occurred but not, on average, passed the 2π/0 point, the adjustment factor remained unchanged. However, when **α**_n_ < **α**_**n-1**_, i.e., growth on average passed the 2π/0 divide within the elapsed timepoint, an adjustment factor of 2π was made to each subsequent timepoint. When this method was applied to in vivo datasets, where sampling variability combined with slow growth rates could lead to spurious negative shifts, we further assumed that average growth rates would not exceed 2.5 generation per hour when interpreting if a population had grown through a full period. Individual *θ*_0_ values were then adjusted by addition of the calculated multiples of 2π. For adjustments, where ${\bar{\alpha}}$ < π, colonies with *θ*_0_ = **α** − π to 0 were assumed to be one phase revolution (i.e., 2π radians) behind of those of 0 to **α + **π. Where **α > **π, colonies with *θ*_0_ = 0 to **α** **−** π were assumed to be one phase revolution ahead of values **α** **−** π to 0. Subsequent calculations treated the adjusted *θ*_0_ values as linear datasets.

To calculate growth on a single-cell level we treated each colony separately, determining the distribution of probability for the generations of growth since the reference timepoint (*t*_sync_) on a single-cell basis. Because adjusted *θ*_0_ values had already been normalized to **α**_**sync**_ = 0 and adjusted for multiple periods of growth where necessary, the mean estimated growth, *μ*, for each individual colony in the population, *i*, is described by:

13 $$\mu _{\it{i}} = \frac{{\theta _{0\left( {{\mathrm{adjusted}}} \right)\,{\it{i}}}}}{{2{\mathrm{\pi }}}} \times {\mathrm{period}}_{{\mathrm{strain}}}$$

where period_strain_ is the calculated mean periodicity of the relevant strain in generations/period.

The distribution of probable growth can then be determined by a combination of the phase distribution of the population at t_sync_ and the uncertainty in our periodicity measurements. Initial synchronized repressilator 2.0 populations (t_sync_) were assumed to be normally distributed, which was appropriate based on visual inspection of Q–Q plots. Thus, the growth probability of each colony, i, is described by a normal distribution with error,σ:

14 $$\sigma _i = \mu _i\sqrt {\left( {\frac{{{\mathrm{period}}\,{\mathrm{error}}}}{{{\mathrm{period}}_{{\mathrm{strain}}}}}} \right)^2 + \left( {\frac{{\sigma _{{\mathrm{tsync}}}}}{{\theta _{0\left( {a{\mathrm{djusted}}} \right){\it{i}}}}}} \right)^2}$$

An empirical probability density function and cumulative distribution function was then calculated as the sum of all individual distributions.

Interpretations of growth data are informed by physiological limits. For example, we can assume that growth will not exceed known growth rates for a given species, and the repressilator can only progress in one direction based on growth. Of the possible outcomes, we report the most consistent growth trajectory across timepoints. An example of how these constraints are applied to a dataset is provided (Supplementary Fig. 6).

### Histograms

For histograms, *θ*_0_ data were separated into π/6-width bins centered on 0 and multiples of π/6. Counts were normalized by the total number of fit colonies in each dataset. Datapoints were plotted at each bin center. For linear histograms, 0 and 2π are plotted as the same value.

### In vivo bacterial growth testing

The Harvard Medical School Animal Care and Use Committee approved all animal study protocols and all experiments complied with relevant ethical regulations.

Female C57Bl/6 (Jackson Laboratory) mice of 8–14 weeks, (including >2 weeks acclimatization to the HMS mouse facility), were used in groups of 2–3 per experiment. Mice were routinely randomized between treatment groups in advance of experiments, where relevant. Mice were fed on a lactose-free chow (Envigo Teklad Global 18% Protein Rodent Diet) for at least 1 week prior to provision of repressilator bacteria to avoid any interruption of the repressilator by lactose. Streptomycin-treated mice were administered 5 mg USP-grade streptomycin sulfate (Gold Biotechnology) in 100 μL sterile PBS by oral gavage, or where specifically stated 0.5 g/L in drinking water supplemented with 5% sucrose overnight. PAS715 *E. coli* MG1655 bacteria or PAS716 *S*. Typhimurium LT2 bacteria were prepared for administration by pelleting from culture, washing and dilution in sterile PBS before provision to mice as a 100μL oral gavage.

In situ synchronization was achieved by provision of USP-grade 10 mM IPTG (Sigma) or 0.1 mg/mL aTc (Sigma) in drinking water supplemented with 5% sucrose overnight.

Fresh fecal samples were collected by temporarily removing mice to small containers until at least 2–3 fecal pellets were produced. Fecal pellets were homogenized in PBS at 50 or 100 mg/mL in sterile PBS by vortexing for ~5 min in 1.5 mL Eppendorf tubes. To remove large debris, homogenized feces was then centrifuged either by briefly pulsing (~1 sec) on a benchtop minifuge (where CFU counts were not critical), or by centrifugation at 4 × *g* for 20 min (where CFU counts were critical). The supernatant was then serially diluted and cultured on selective agar plates.

For inflammation experiments, mice were fed Dextran Sulfate Sodium (Colitis Grade, M.W. = 36,000–50,000, MP Biomedicals, LLC) in drinking water supplemented with 5% sucrose for 3 days prior to bacterial administration. DSS water was exchanged every second day, and mice remained exposed throughout the course of the experiment.

LCN-2 biomarker levels^50^ were quantified as previously^36^ using a Mouse lipocalin-2/NGAL DuoSet ELISA kit (R&D Systems). Fecal pellets were vortexed at 100 mg/mL in PBS+0.1% Tween20 for 20 min, then pelleted at 12,000 × *g* for 10 min at 4 °C. ELISA results were obtained on a BioTek Synergy H1 plate reader. Four hundred fifty nanometer absorbance corrected values were interpolated from a sigmoidal four parameter logistic standard curve using Prism 7 for Mac OSX software (GraphPad).

### Peak-to-trough ratio analysis

Peak-to-trough ratios were computed from metagenomic sequencing of mouse fecal samples as previously described^15^. Genomic DNA was extracted from flash-frozen fecal samples using a Qiagen DNEasy Blood & Tissue Kit. Genomic DNA from each sample was prepared for sequencing using a Kapa HyperPlus Kit and sequenced using an Illumina NextSeq at the Bauer Core Facility at Harvard University. Sequencing reads were trimmed for quality using Trimmomatic 0.36^51^ and each sample was aligned to the genome of interest using BWA mem 0.7.8^52^. Sequencing coverage was obtained using bedtools 2.27.1^53^. To calculate peak-to-trough ratio, mean coverage was computed for 10 kb bins across the genome and a segmented linear model was fit to the coverage using the R package segmented^54^.

### Growth in the mother machine

We performed time-lapse single-cell measurements to monitor and compare the dynamics of repressilator circuits in rich defined medium (EZ Rich Defined Medium; Teknova) and in cecum contents. Cecum contents were derived from fresh or frozen mouse cecum, diluted in PBS. 2–4 FVB (Charles River) or C57/Bl6 (Jackson) mice were sacrificed, their cecum dissected, and the cecum contents were extracted by scraping and washing off the tissue with PBS. When frozen, cecum contents were placed at −80 °C. The contents were then thawed, if necessary, and diluted in PBS, spun briefly in a benchtop centrifuge (13,000 × *g*) to remove large particulate matter and the supernatant removed and saved. Cecum matter was then washed a second time, centrifuged, and the supernatant pooled with the first wash. Pooled supernatant was centrifuged a second time, then passed sequentially through a 20 μm syringe filter (Millex-AP glass fiber 25 mm syringe filter) and then a 5 μm syringe filter (Pall Acrodisc 32 mm syringe filter with 5 μm Supor^®^ membrane). The filtered fluid was then used as an input for the mother machine.

The filtered fluid from the cecum contents was loaded in 10 mL syringes and pumped through mother-machine devices^32^ using a Higher Pressure Programmable Syringe Pump (NE1000). To flow the cecum through the device, we used a mother-machine design where the dimensions of the flow channel has been optimized for flowing dense cultures and maintaining low pressure to allow low fluid flow-rates (Bakshi, S., Leoncini, E., Baker, C., Cañas-Duarte, S.J., Okumus, B. and Paulsson, J., personal communication). Device fabrication used a wafer prepared using standard UV photolithography, with quartz-chrome photomasks (Toppan Inc) in a clean-room environment. Devices consisted of three independent layers: a Su8 ‘base’ coat, a featureless uniform layer of completely cured Su8; cell channels (25 μm long, 1.5 μm wide, and 1.3 μm height) placed in the orthogonal direction to the flow channels; and medium flow channels, of 150 μm width and 45 μm height.

The devices were constructed from the wafer using soft lithographic techniques in clean-room (Harvard Medical School Microfabrication Core). Dimethyl siloxane monomer (Sylgard 184) was mixed with curing agent (10:1 ratio), defoamed, poured onto the silicon wafer, degassed (1 h) and cured at 65°C (1 h). Individual chips were cut and the inlets and outlets punched with a biopsy puncher. On the day of experiments, bonding to KOH-cleaned cover slips was done using oxygen plasma treatment (30 sec at 50 W and O_2_ pressure at 170 mTorr). Chips were incubated at 95 °C, >30 min to reinforce bonding before being cooled to room-temperature.

Cell channels were loaded with E. coli containing the repressilator circuit. In some experiments *E. coli* PAS718, constitutively expressing mKate2, was used, with the mKate2 acting as a marker to segment single cells and to extract intensities and cell-growth estimates more effectively. We used a slow flow-settings (5 μL/min) to ensure we could observe the dynamics of the cells for prolonged periods (>10 h) with the small volume of cecum contents (~3–5 mL). This ensured that we observed at least two peaks for repressilator signals in individual channels (YFP or CFP) for a majority of the cells, which is necessary to calculate the period of oscillation. To minimize phototoxicity the frequency of imaging was kept at 6 min/frame. This gives about 4–5 snapshots per generation time, which is enough to get a good estimate of growth rate and also a smooth intensity time-series. For *E. coli* PAS715 experiments imaging occurred at 10 min/frame.

Images were acquired using a Nikon Ti inverted microscope equipped with a temperature-controlled incubator (OKO lab), a sCMOS camera (ANDOR), a 40X Plan Apo air objective (NA 0.95, Nikon), an automated xy-stage (Nikon) and light engine LED excitation source (Lumencor SpectraX). All experiments were performed at 37 °C. Microscope control was done with Nikon Elements software. We acquired time-lapse data from 40 fields of view, which allows us to track approximately 2000 cells simultaneously. The acquired data were analyzed using a hybrid analysis platform written in the Paulsson lab. In brief, images were segmented using a custom-designed FIJI plugin and then the extracted data were further processed to track cells in a custom-designed MATLAB script. Time-series of cell-size data calculated from the segmented images was used to compute generation times. The time-series of YFP channel intensity was used to calculate the period of repressilator in the cecum content.

### Figure generation

Figures were generated through Adobe Illustrator CC2018 and CC2019, Adobe InDesign CC2018 and CC2019, Prism v6-7 (Graphpad), and MATLAB versions R2017a-2018a (Mathworks). Many figures utilize color palettes based on research by Cynthia Brewer^55^.

## Supplementary information

Supplementary Information

Peer Review File

Source Data

## Data Availability

Sequence reads for peak-to-trough ratio analyses are available at NCBI SRA (accession: PRJNA542389 [https://www.ncbi.nlm.nih.gov/sra/PRJNA542389]). All plasmids used in the study are available from Addgene, with identification numbers as follows: pLPT234 (# 127855); pLPT41 (#85524); pLPT145 (#85527); pLPT149 (#85529). All other data or resources are available in the Source Data file or from the corresponding author upon reasonable request.

## Code availability

Core code used for RINGS analysis is available at Github (https://github.com/hms-idac/repressilator-colony-rings).
