# Author Correction: Bacterial variability in the mammalian gut captured by a single-cell synthetic oscillator

**DOI:** 10.1038/s41467-021-22149-5

**Published:** 2021-03-16

**Authors:** David T. Riglar, David L. Richmond, Laurent Potvin-Trottier, Andrew A. Verdegaal, Alexander D. Naydich, Somenath Bakshi, Emanuele Leoncini, Lorena G. Lyon, Johan Paulsson, Pamela A. Silver

**Affiliations:** 1 Department of Systems Biology, Harvard Medical School, Boston, MA USA; 2 Wyss Institute for Biologically Inspired Engineering, Harvard University, Boston, MA USA; 3 Image and Data Analysis Core, Harvard Medical School, Boston, MA USA; 4 Harvard John A. Paulson School of Engineering and Applied Sciences, Cambridge, MA USA; 5 Present Address: Department of Infectious Disease, Imperial College London, London, UK; 6 Present Address: Biology Department, Concordia University, Montreal, QC Canada; 7 Present Address: Department of Engineering, Cambridge University, Cambridge, UK

**Keywords:** Synthetic biology, Cell division, Bacterial host response, Oscillators

Correction to: *Nature Communications* 10.1038/s41467-019-12638-z, published online 11 October 2019.

In the original version of the Supplementary Information provided with this Article, Supplementary Table 1 incorrectly provided the details for strain LPT239 as “*E. coli* MC4100 + pLPT234 + pLPT145” and for strain LPT320 as “*E. coli* MC4100 + pLPT234 + pLPT41”. The details for strain LPT239 should be “*E. coli* MC4100 + pLPT234 + pLPT41” and for strain LPT320 should be “*E. coli* MC4100 + pLPT234 + pLPT145”. This has been corrected in the HMTL version of the article and the correct table appended below:

Original

**Supplementary Table 1:** Strains used in this study.

**Table:** 

Strain	Source	Details
LPT239	This study	*E. coli* MC4100 + pLPT234 + pLPT145
LPT320	This study	*E. coli* MC4100 + pLPT234 + pLPT41
LPT322	This study	*E. coli* MC4100 + pLPT234 + pLPT149
PAS715	This study	*E. coli* MG1655 DE(*lacI*) DE(*motA*) *rpsL* K42R + pLPT234 + pLPT145
PAS716	This study	*S*. Typhimurium LT2 + pLPT234 + pLPT145 (*streptomycin resistant through uncharacterized mutational selection).
PAS717	This study	*E. coli* Nissle 1917 DE(*lacI*) DE(*motA*) *rpsL* K42R + pLPT234 + pLPT145
PAS718	This study	*E. coli* MG1655 DE(*lacI*) DE(*motA*) *rpsL* K42R attTn7::pRNA1-mKate2 + pLPT234 + pLPT145

Corrected:

**Supplementary Table 1:** Strains used in this study.

**Table:** 

Strain	Source	Details
LPT239	This study	*E. coli* MC4100 + pLPT234 + pLPT41
LPT320	This study	*E. coli* MC4100 + pLPT234 + pLPT145
LPT322	This study	*E. coli* MC4100 + pLPT234 + pLPT149
PAS715	This study	*E. coli* MG1655 DE(*lacI*) DE(*motA*) *rpsL* K42R + pLPT234 + pLPT145
PAS716	This study	*S*. Typhimurium LT2 + pLPT234 + pLPT145 (*streptomycin resistant through uncharacterized mutational selection).
PAS717	This study	*E. coli* Nissle 1917 DE(*lacI*) DE(*motA*) *rpsL* K42R + pLPT234 + pLPT145
PAS718	This study	*E. coli* MG1655 DE(*lacI*) DE(*motA*) *rpsL* K42R attTn7::pRNA1-mKate2 + pLPT234 + pLPT145

## Supplementary information

Supplementary Materials

