# Supplementary material for: Bacterial variability in the mammalian gut captured by a single-cell synthetic oscillator
Source: Nat Commun. 2019 Oct 11;10:4665. doi: 10.1038/s41467-019-12638-z (PMC6789134; doi:10.1038/s41467-019-12638-z)
Supplement: Supplementary file 1 — Supplementary Information [file 41467_2019_12638_MOESM1_ESM.pdf]

SUPPLEMENTARY MATERIALS FOR:

Bacterial variability in the mammalian gut captured by a single-cell synthetic oscillator

Authors:
David T Riglar [1, 2]†, David L Richmond [3*], Laurent Potvin-Trottier [1] ‡ [*], Andrew A Verdegaal [1], Alexander D Naydich [1,2,4], Somenath Bakshi [1] ^, Emanuele Leoncini [1], Lorena G Lyon [1], Johan Paulsson [1] & Pamela A Silver [1, 2].

Affiliations:
[1] Department of Systems Biology, Harvard Medical School, Boston MA, USA
[2] Wyss Institute for Biologically Inspired Engineering, Harvard University, Boston MA, USA
[3] Image and Data Analysis Core, Harvard Medical School Boston MA, USA
[4] Harvard John A. Paulson School of Engineering and Applied Sciences, Cambridge MA

Correspondence to Pamela_Silver@hms.harvard.edu

† Present address: Department of Infectious Disease, Imperial College London, London, UK
‡ Present address: Biology Department, Concordia University, Montreal QC, Canada
^ Present address: Department of Engineering, Cambridge University, Cambridge, UK
[*]Authors contributed equally

CONTENTS:

Supplementary Figures 1-10.

Supplementary Tables 1-3

Supplementary References

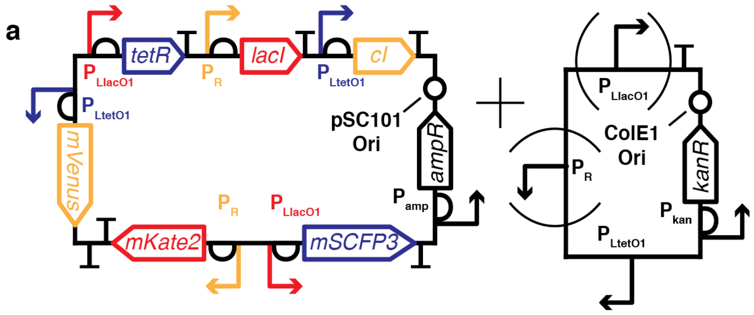

Supplementary Figure 1. The repressilator 2.0 plasmids
A. Schematics of the triple-reporter repressilator and sponge plasmids used throughout the study.

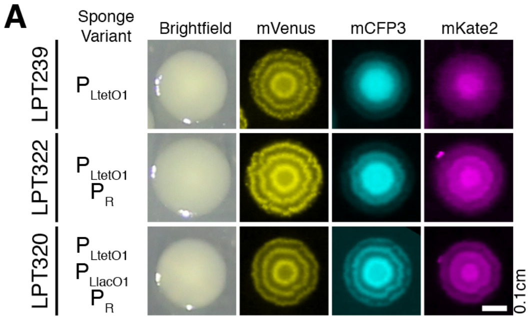

B

	Sponge Variant	oscillations visible in test colonies: mVenus	mCFP3	mKate2
LPT239	P_{LtetO1}	70/72	1/72	1/72
LPT322	P_{LtetO1} P_R	96/96	96/96	59/96
LPT320	P_{LtetO1} P_{LlacO1} P_R	120/120	120/120	120/120

Supplementary Figure 2: Sponge plasmid variation allows multicolor RINGS analysis
A. Fluorescent imaging of colonies formed by *E. coli* MC4100 bacterial carrying the repressilator 2.0 along with sponge plasmid variants - P_{LtetO1} only (LPT239), P_{LtetO1} + P_R (LPT322) and P_{LtetO1} + P_R + P_{LlacO1} (LPT320) - showed variability in the consistency of fluorescent rings formed. Scale bar = 0.1cm **B.** Visual inspection of colonies from each strain also showed the clear presence of fluorescent rings differed across each population.

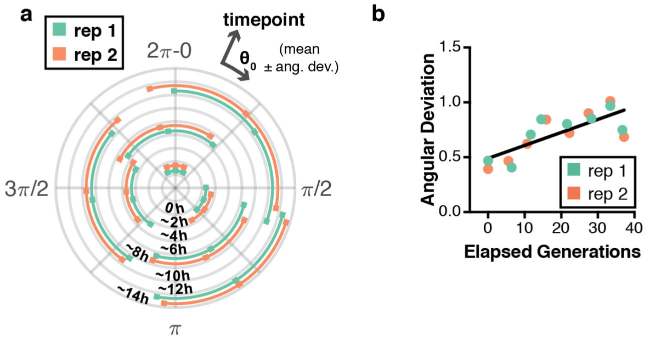

Supplementary Figure 3: Angular deviation change of LPT320 during growth.

A. Summary data from Fig 1 G-J. Graph shows circular mean ± angular deviation (angle) of RINGS measured phase (θ_0) for two biological replicates. Each ring in the graph corresponds to a discrete timepoint, taken every 2h ± 15min. **B.** Angular deviation of LPT320 throughout the experiment.

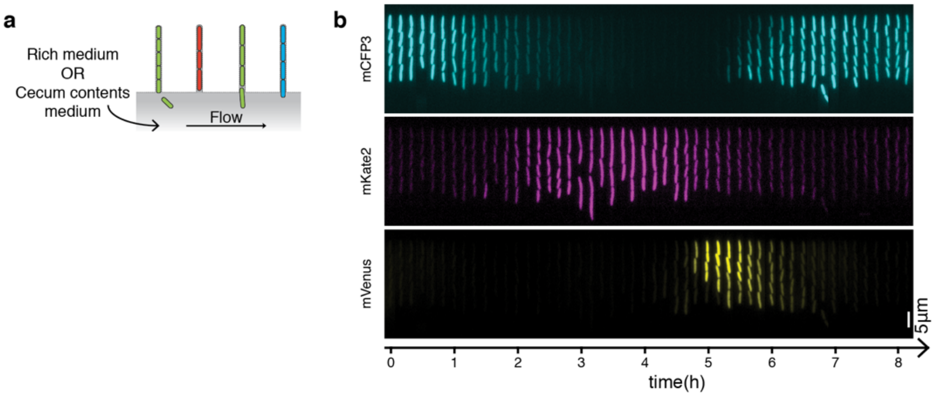

Supplementary Figure 4. Repressilator 2.0 period calculation using single-cell imaging in a microfluidic device.

A. The mother machine is a microfluidic device consisting of trenches that can be seeded with individual bacterial lineages, arranged around a central flow channel that delivers growth medium to the cells. **B.** Kymograph of fluorescent timelapse images from a single growth trench demonstrates oscillation of the repressilator 2.0 in PAS715 bacteria during growth on mouse cecum contents medium. Scale bar = 5µm.

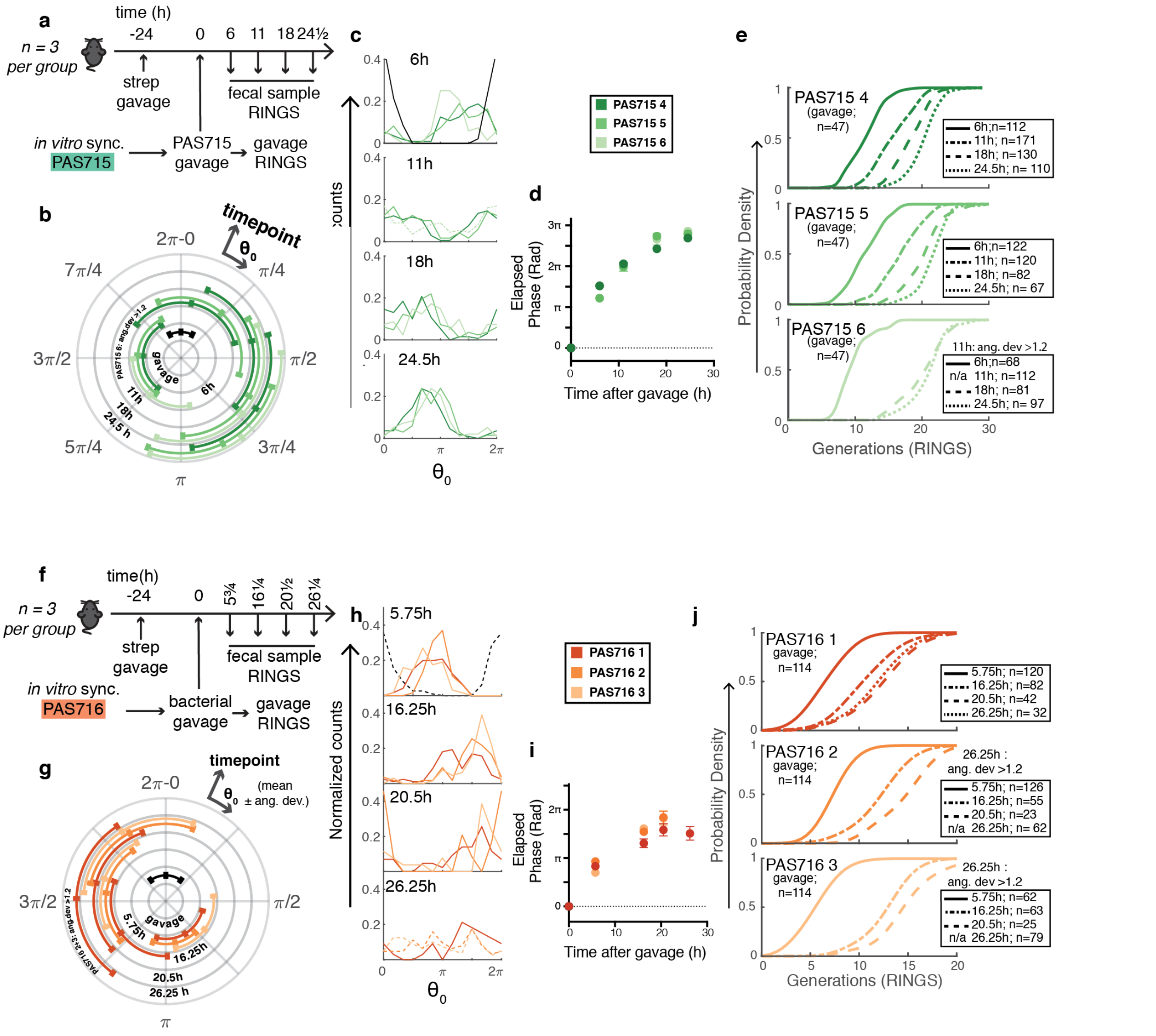

Supplementary Figure 5. The repressilator 2.0 is robust and informative within the mouse gut

A. *in vitro* synchronized PAS715 (*E. coli* MG1655) bacteria measure growth in mice treated with a single dose of streptomycin (n=3 per group). **B.** Repressilator phase progressed throughout the experiment. Graph shows circular mean ± angular deviation (angle) of RINGS measured phase (θ_0). Each ring in the graph corresponds to a discrete timepoint. For number of colonies analyzed at each timepoint in B-E, see corresponding data in E. **C.** Histograms of bacterial phase distributions throughout the experiment. Graphs show normalized counts. 6h timepoint is compared to the gavage population (black line). Dotted lines represent datasets with angular deviation >1.2, which were excluded from downstream analyses. **D.** Graph of average elapsed phase of the population vs time for each mouse. Graph shows mean with 95% CI. **E.** Cumulative distribution functions of predicted generations elapsed since gavage. **F.** *in vitro* synchronized PAS716 (*S.* Typhimurium LT2) bacteria measure growth in mice treated with a single dose of streptomycin (n=3 per group). **G.** Repressilator phase progressed throughout the experiment. Graph shows circular mean ± angular deviation (angle) of RINGS measured phase

(θ_0). Each ring in the graph corresponds to a discrete timepoint. For number of colonies analyzed at each timepoint for G-J, see corresponding data in J. **H.** Histograms of bacterial phase distributions throughout the experiment. Graphs show normalized counts. 5.75h timepoint is compared to the gavage population (black line). Dotted lines represent datasets with angular deviation >1.2, which were excluded from downstream analyses. **I.** Graph of average elapsed phase of the population vs time for each mouse. Graph shows mean with 95% CI. **J.** Cumulative distribution functions of predicted generations elapsed since gavage. Source data are provided as a Source Data file.

a Key constraints:

- Growth cannot be negative
- Growth in the gut will not exceed 2.5 gen/hour (the strain's growth in ideal aerobic conditions), and will likely be considerably slower in the anaerobic environment.
- Growth rate will most likely follow a smooth trajectory between adjacent timepoints and be consistent across replicates.

b

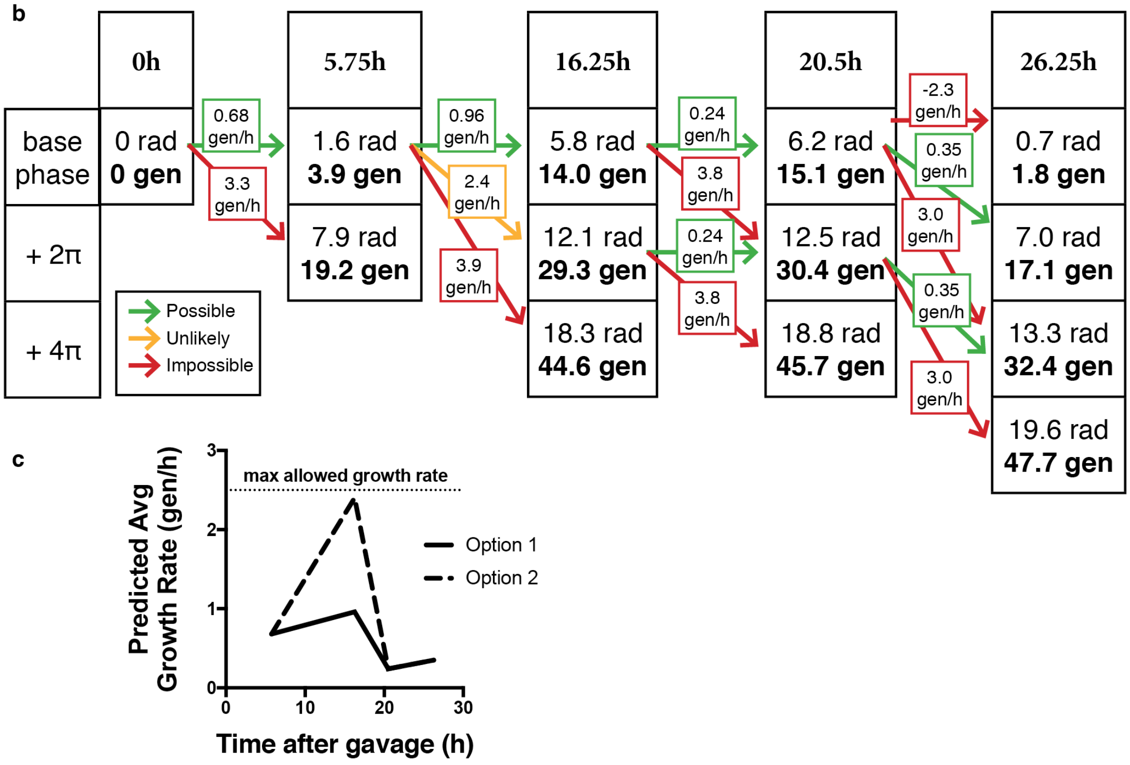

Supplementary Figure 6. Example decision matrix for determination of elapsed phase and growth using the RINGS analysis method.

A. Due to physiological limitations of bacterial growth and the repressilator circuit, a set of key constraints can be used to assist in confidently assigning growth estimates using RINGS-derived phase values. **B.** In particular, RINGS-based phase data was assessed for the potential that >1 period of the oscillator had elapsed between timepoints. Data shown is derived from sample PAS715 1 (Fig 4). Population average values for phase are displayed as the modulo-2π "base" phase, and +2π increments (ie. +2π assumes an additional full period of growth has elapsed). Arrows are labeled with the associated growth rate calculation for progression between two timepoint-modulo variants to have occurred, with color depicting those that fall within (green), outside of (red), or at the limits of our growth constraints (yellow). **C.** Possible growth attributions were also assessed across adjacent timepoints of single experiments. Growth is expected to maintain a level of consistency between timepoints, assisting in assignment of the most likely growth elapsed (eg. option 1). Graph shows growth rates calculated in B at each timepoint for the PAS715 1 dataset. Max allowed growth rate line corresponds to the growth of the strain under ideal laboratory conditions of 2.5 gen/h.

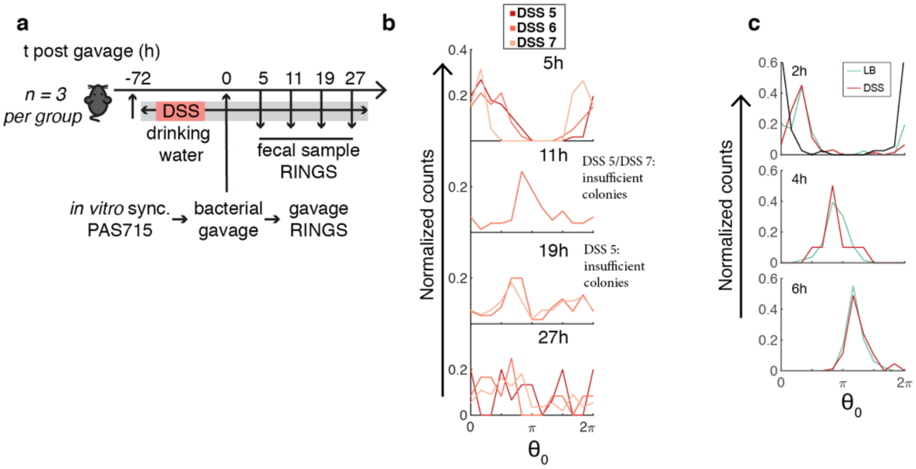

Supplementary Figure 7: RINGS identifies increased growth variability in the inflamed gut
A. RINGS was used to analyze PAS715 growth in the dextran sulfate sodium (DSS) inflamed mouse gut. **B.** Histograms of normalized bacterial phase counts in fecal samples. Graphs show normalized counts. Numbers are as follows: DSS5/6/7 – 5h: 56/100/19. 11h: -/75/-. 19h: -/ 55/50. 27h: 15/12/55. **C.** RINGS analysis of PAS715 following *in vitro* growth in LB ± 4% DSS. Graphs show normalized counts. Numbers are as follows: 0h: 37. LB control - 2h:62. 4h:10. 6h:88. LB+DSS - 2h:41. 4h:56. 6h. 49. Source data are provided as a Source Data file.

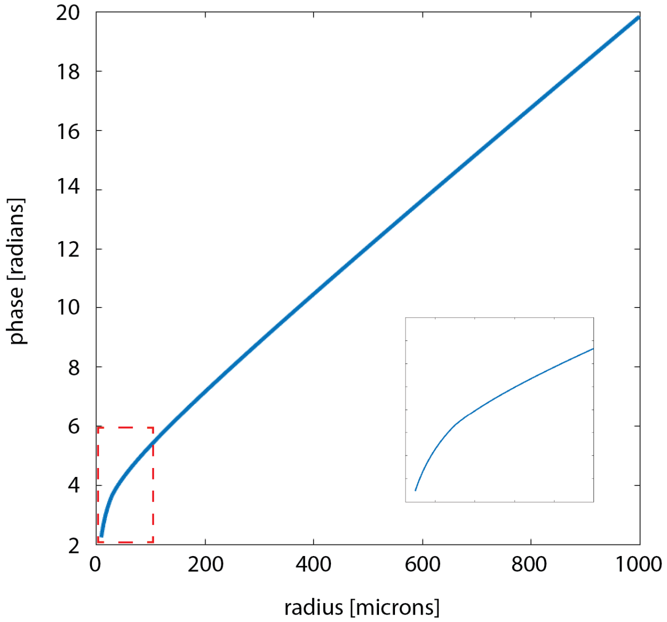

Supplementary Figure 8: A model for bacterial colony growth.
Phase profile of a combined growth model based on an exponential growth phase at low radius and linear growth phase at high radius. Inset shows region marked in red.

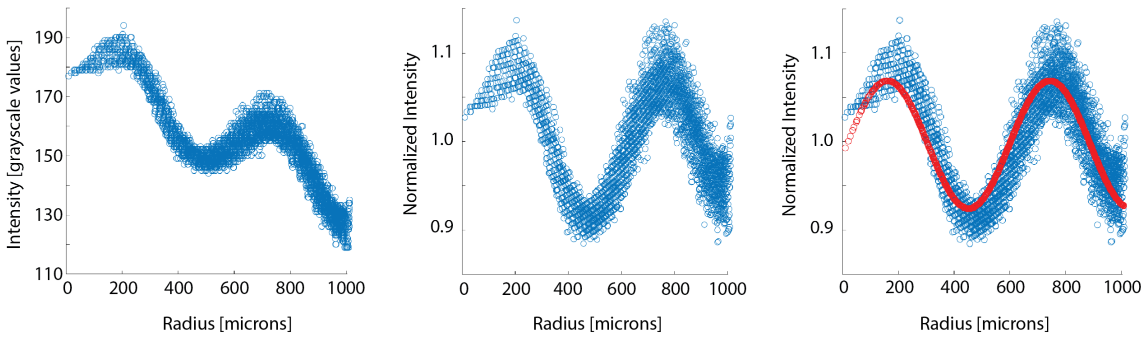

Supplementary Figure 9: Example of data normalization and curve fitting by RINGS.
A. The raw intensity values from the image of a single colony, demonstrating intensity loss at increasing radius. **B.** Intensity values after normalizing by a second order polynomial model. **C.** The model fit (red) can be seen overlaid on raw data (blue).

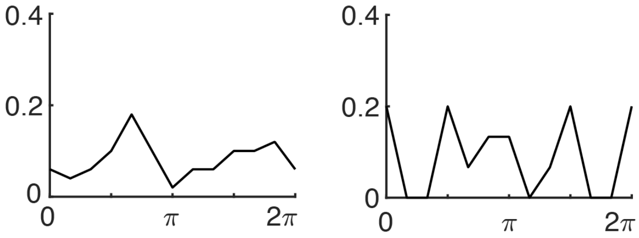

Supplementary Figure 10: Examples of datasets with angular deviation >1.2
Datasets with angular deviation >1.2 were routinely removed from downstream analyses of elapsed phase due to reduced confidence in the accuracy of circular mean calculations and the classification of colonies close to the circular mean ± π divide.

Supplementary Table 1: Strains used in this study.

Strain	Source	Details
LPT239	This study	*E. coli* MC4100 + pLPT234 + pLPT145
LPT320	This study	*E. coli* MC4100 + pLPT234 + pLPT41
LPT322	This study	*E. coli* MC4100 + pLPT234 + pLPT149
PAS715	This study	*E. coli* MG1655 DE(*lacI*) DE(*motA*) *rpsL* K42R + pLPT234 + pLPT145
PAS716	This study	*S.* Typhimurium LT2 + pLPT234 + pLPT145 (*streptomycin resistant through uncharacterized mutational selection).
PAS717	This study	*E. coli* Nissle 1917 DE(*lacI*) DE(*motA*) *rpsL* K42R + pLPT234 + pLPT145
PAS718	This study	*E. coli* MG1655 DE(*lacI*) DE(*motA*) *rpsL* K42R attTn7::pRNA1-mKate2 + pLPT234 + pLPT145

Supplementary Table 2: Peak-to-Trough ratio values. PTR as calculated from analysis of metagenomic samples sequenced by Illumina sequencing. Higher values correspond to higher estimated growth rates.

Bacterial strain	mouse	Peak-to-Trough Ratio			
		Pre-admin	5.75h	16.25h	20.5h
PAS715	1	<5% map	1.65	1.43	1.46
	3	<5% map	1.75	1.40	1.52
PAS716	1	<5% map	N/A	1.55	N/A
	2	<5% map	1.83	1.43	N/A
	3	<5% map	1.66	1.36	N/A

Supplementary Table 3: Plasmids used in this study

Plasmid name	Source	Details
pLPT234	This study	pSC101, Amp, repressilator 2.0 plasmid P_{LtetO1}-*cI*, P_R-*lacI*, P_{LlacO1}-*tetR*, P_{LtetO1}-*venus*, P_{LlacO1}-*cfp*, P_R-*mKate2*
pLPT41	[1]	ColE1, Kan, P_{tetO1} sponge plasmid
pLPT149	[1]	ColE1, Kan, P_{tetO1}+P_R sponge plasmid
pLPT145	[1]	ColE1, Kan, P_{tetO1}+P_R+P_{lacO1} sponge plasmid

Supplementary References

1. Potvin-Trottier L, Lord ND, Vinnicombe G, Paulsson J. Synchronous long-term oscillations in a synthetic gene circuit. *Nature* **538**, 514-517 (2016).
