# Supplementary material for: Bacterial variability in the mammalian gut captured by a single-cell synthetic oscillator
Source: Nat Commun. 2019 Oct 11;10:4665. doi: 10.1038/s41467-019-12638-z (PMC6789134; doi:10.1038/s41467-019-12638-z)
Supplement: Supplementary file 2 — Peer Review File [file 41467_2019_12638_MOESM2_ESM.pdf]

Editorial Note: This manuscript has been previously reviewed at another journal that is not operating a transparent peer review scheme. This document only contains reviewer comments and rebuttal letters for versions considered at *Nature Communications*. Mentions of the other journal have been redacted.
