# Supplementary material for: Author Correction: Bacterial variability in the mammalian gut captured by a single-cell synthetic oscillator
Source: Nat Commun. 2021 Mar 16;12:1818. doi: 10.1038/s41467-021-22149-5 (PMC7966751; doi:10.1038/s41467-021-22149-5)
Supplement: Supplementary file 1 — Supplementary Materials [file 41467_2021_22149_MOESM1_ESM.pdf]

SUPPLEMENTARY MATERIALS FOR:

Bacterial variability in the mammalian gut captured by a single-cell synthetic oscillator

Authors:
David T Riglar [1, 2]†, David L Richmond [3*], Laurent Potvin-Trottier [1] ‡ [*], Andrew A Verdegaal [1], Alexander D Naydich [1,2,4], Somenath Bakshi [1] ^, Emanuele Leoncini [1], Lorena G Lyon [1], Johan Paulsson [1] & Pamela A Silver [1, 2].

Affiliations:
[1] Department of Systems Biology, Harvard Medical School, Boston MA, USA
[2] Wyss Institute for Biologically Inspired Engineering, Harvard University, Boston MA, USA
[3] Image and Data Analysis Core, Harvard Medical School Boston MA, USA
[4] Harvard John A. Paulson School of Engineering and Applied Sciences, Cambridge MA

Correspondence to Pamela_Silver@hms.harvard.edu

† Present address: Department of Infectious Disease, Imperial College London, London, UK
‡ Present address: Biology Department, Concordia University, Montreal QC, Canada
^ Present address: Department of Engineering, Cambridge University, Cambridge, UK
[*]Authors contributed equally

CONTENTS:

Supplementary Figures 1-10.

Supplementary Tables 1-3

Supplementary References

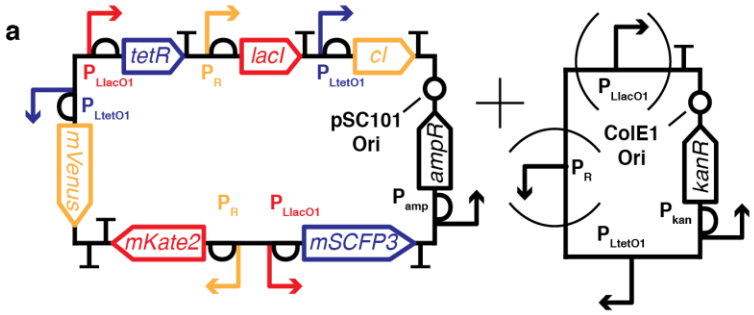

Supplementary Figure 1. The repressilator 2.0 plasmids
A. Schematics of the triple-reporter repressilator and sponge plasmids used throughout the study.

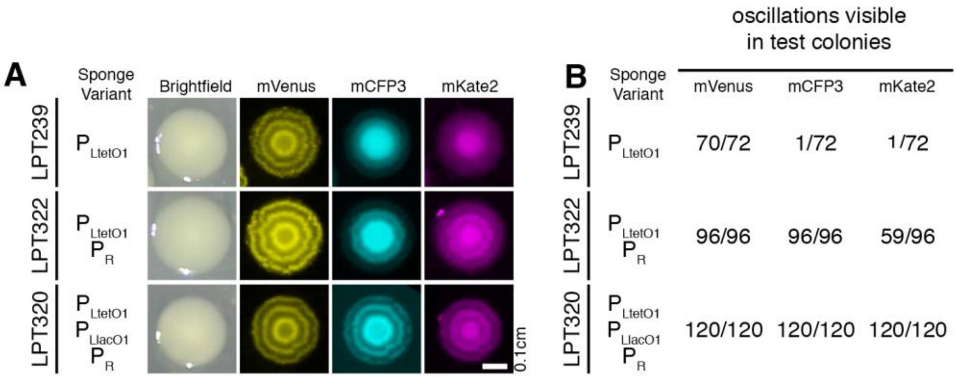

Supplementary Figure 2: Sponge plasmid variation allows multicolor RINGS analysis
A. Fluorescent imaging of colonies formed by *E. coli* MC4100 bacterial carrying the repressilator 2.0 along with sponge plasmid variants - P_{LtetO1} only (LPT239), P_{LtetO1} + P_R (LPT322) and P_{LtetO1} + P_R + P_{LlacO1} (LPT320) - showed variability in the consistency of fluorescent rings formed. Scale bar = 0.1cm **B.** Visual inspection of colonies from each strain also showed the clear presence of fluorescent rings differed across each population.

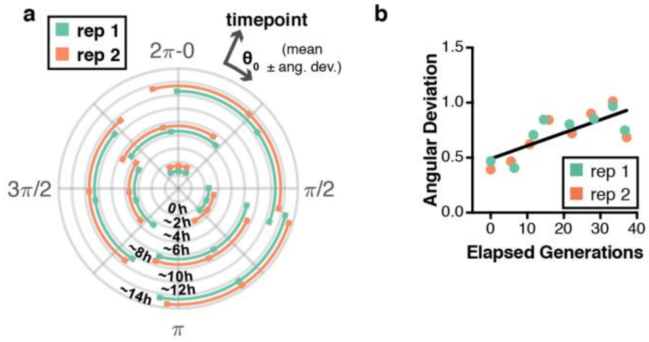

Supplementary Figure 3: Angular deviation change of LPT320 during growth.
A. Summary data from Fig 1 G-J. Graph shows circular mean ± angular deviation (angle) of RINGS measured phase (θ_0) for two biological replicates. Each ring in the graph corresponds to a discrete timepoint, taken every 2h ± 15min. **B.** Angular deviation of LPT320 throughout the experiment.

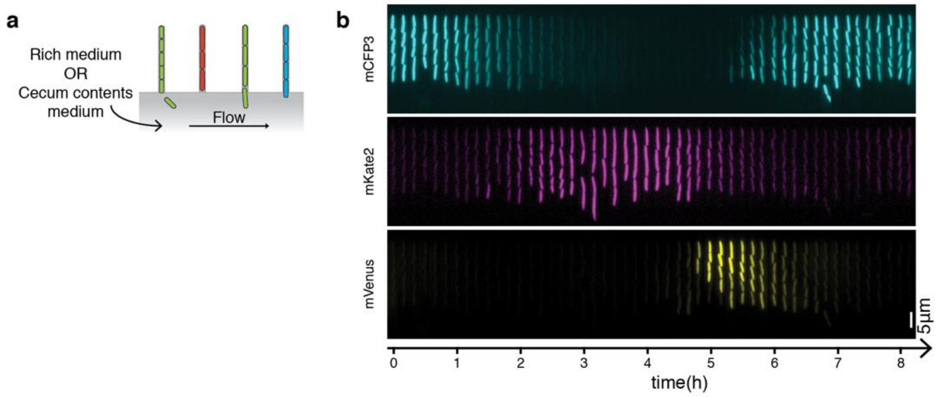

Supplementary Figure 4. Repressilator 2.0 period calculation using single-cell imaging in a microfluidic device.

A. The mother machine is a microfluidic device consisting of trenches that can be seeded with individual bacterial lineages, arranged around a central flow channel that delivers growth medium to the cells. **B.** Kymograph of fluorescent timelapse images from a single growth trench demonstrates oscillation of the repressilator 2.0 in PAS715 bacteria during growth on mouse cecum contents medium. Scale bar = 5μm.

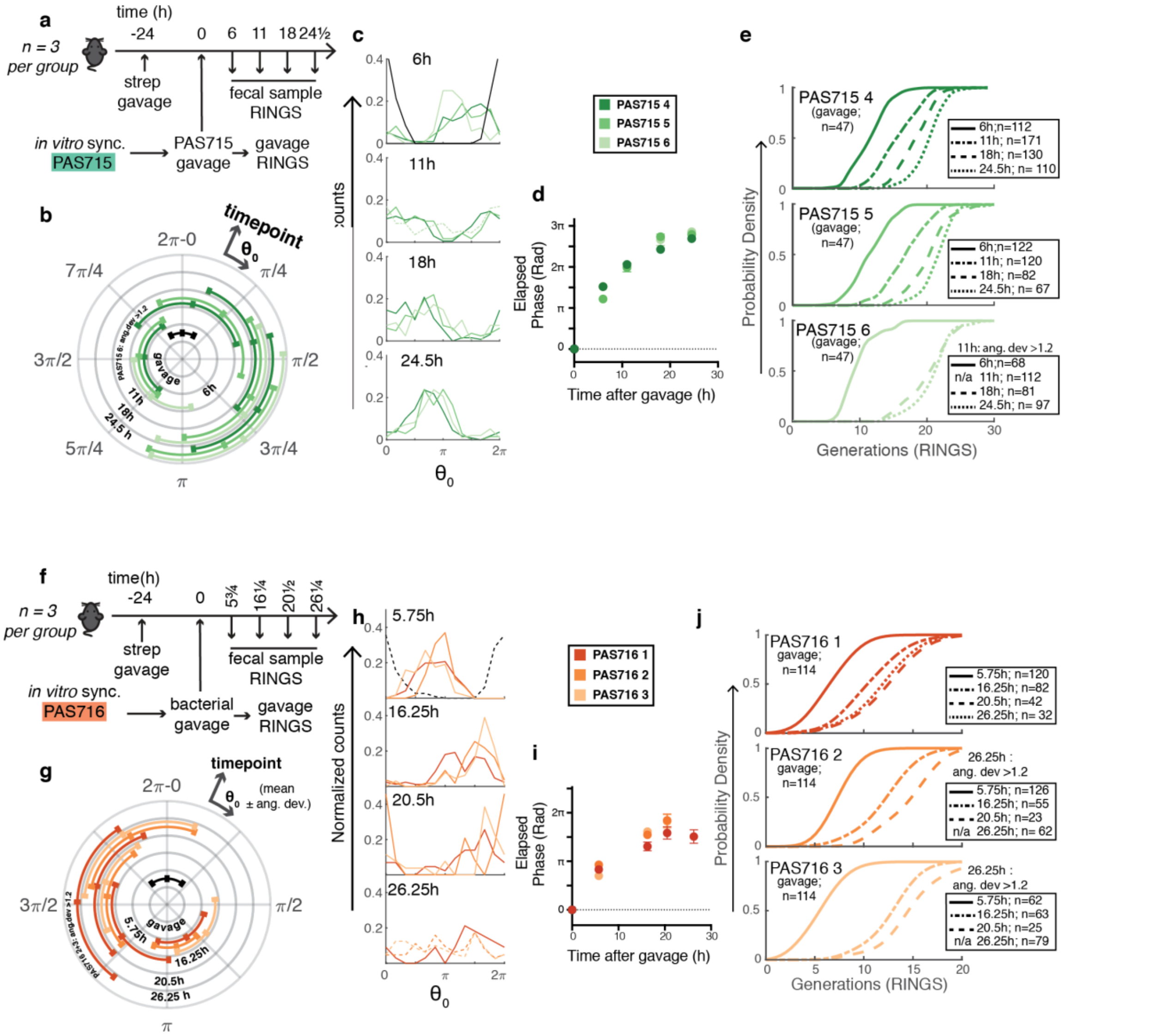

Supplementary Figure 5. The repressilator 2.0 is robust and informative within the mouse gut

A. *in vitro* synchronized PAS715 (*E. coli* MG1655) bacteria measure growth in mice treated with a single dose of streptomycin (n=3 per group). **B.** Repressilator phase progressed throughout the experiment. Graph shows circular mean ± angular deviation (angle) of RINGS measured phase (θ_0). Each ring in the graph corresponds to a discrete timepoint. For number of colonies analyzed at each timepoint in B-E, see corresponding data in E. **C.** Histograms of bacterial phase distributions throughout the experiment. Graphs show normalized counts. 6h timepoint is compared to the gavage population (black line). Dotted lines represent datasets with angular deviation >1.2, which were excluded from downstream analyses. **D.** Graph of average elapsed phase of the population vs time for each mouse. Graph shows mean with 95% CI. **E.** Cumulative distribution functions of predicted generations elapsed since gavage. **F.** *in vitro* synchronized PAS716 (*S.* Typhimurium LT2) bacteria measure growth in mice treated with a single dose of streptomycin (n=3 per group). **G.** Repressilator phase progressed throughout the experiment. Graph shows circular mean ± angular deviation (angle) of RINGS measured phase

(θ_0). Each ring in the graph corresponds to a discrete timepoint. For number of colonies analyzed at each timepoint for G-J, see corresponding data in J. **H.** Histograms of bacterial phase distributions throughout the experiment. Graphs show normalized counts. 5.75h timepoint is compared to the gavage population (black line). Dotted lines represent datasets with angular deviation >1.2, which were excluded from downstream analyses. **I.** Graph of average elapsed phase of the population vs time for each mouse. Graph shows mean with 95% CI. **J.** Cumulative distribution functions of predicted generations elapsed since gavage. Source data are provided as a Source Data file.

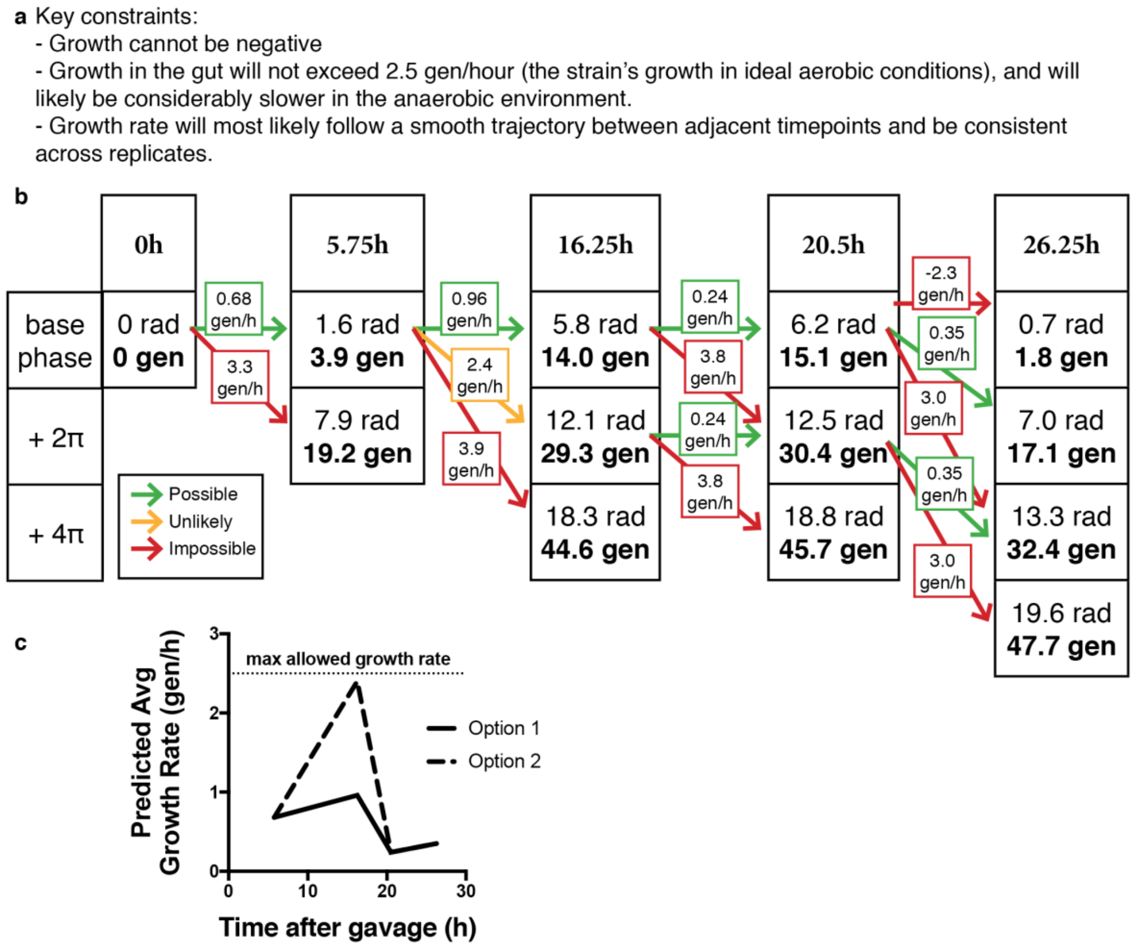

Supplementary Figure 6. Example decision matrix for determination of elapsed phase and growth using the RINGS analysis method.

A. Due to physiological limitations of bacterial growth and the repressilator circuit, a set of key constraints can be used to assist in confidently assigning growth estimates using RINGS-derived phase values. **B.** In particular, RINGS-based phase data was assessed for the potential that >1 period of the oscillator had elapsed between timepoints. Data shown is derived from sample PAS715 1 (Fig 4). Population average values for phase are displayed as the modulo-2π "base" phase, and +2π increments (ie. +2π assumes an additional full period of growth has elapsed). Arrows are labeled with the associated growth rate calculation for progression between two timepoint-modulo variants to have occurred, with color depicting those that fall within (green), outside of (red), or at the limits of our growth constraints (yellow). **C.** Possible growth attributions were also assessed across adjacent timepoints of single experiments. Growth is expected to maintain a level of consistency between timepoints, assisting in assignment of the most likely growth elapsed (eg. option 1). Graph shows growth rates calculated in B at each timepoint for the PAS715 1 dataset. Max allowed growth rate line corresponds to the growth of the strain under ideal laboratory conditions of 2.5 gen/h.

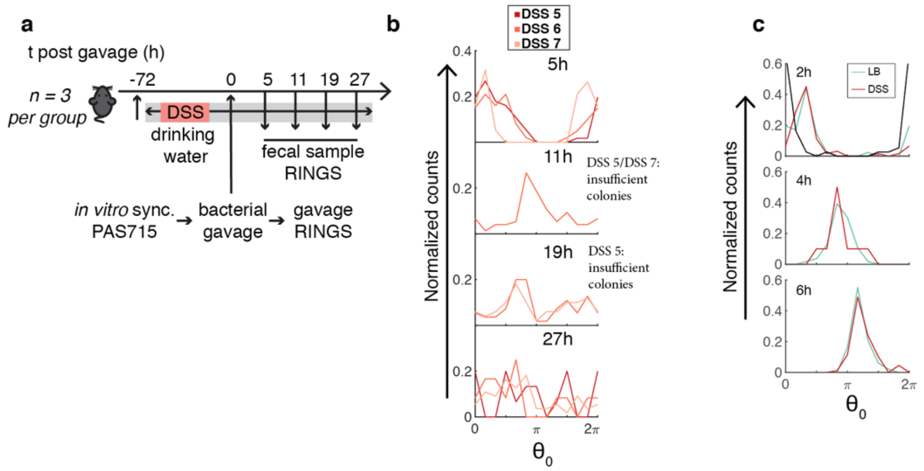

Supplementary Figure 7: RINGS identifies increased growth variability in the inflamed gut
A. RINGS was used to analyze PAS715 growth in the dextran sulfate sodium (DSS) inflamed mouse gut. **B.** Histograms of normalized bacterial phase counts in fecal samples. Graphs show normalized counts. Numbers are as follows: DSS5/6/7 – 5h: 56/100/19. 11h: -/75/-. 19h: -/ 55/50. 27h: 15/12/55. **C.** RINGS analysis of PAS715 following *in vitro* growth in LB ± 4% DSS. Graphs show normalized counts. Numbers are as follows: 0h: 37. LB control - 2h:62. 4h:10. 6h:88. LB+DSS - 2h:41. 4h:56. 6h. 49. Source data are provided as a Source Data file.

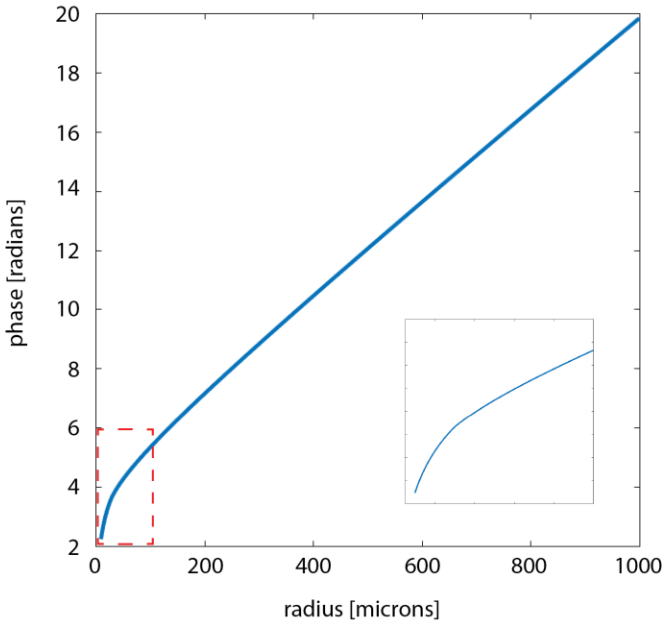

Supplementary Figure 8: A model for bacterial colony growth.

Phase profile of a combined growth model based on an exponential growth phase at low radius and linear growth phase at high radius. Inset shows region marked in red.

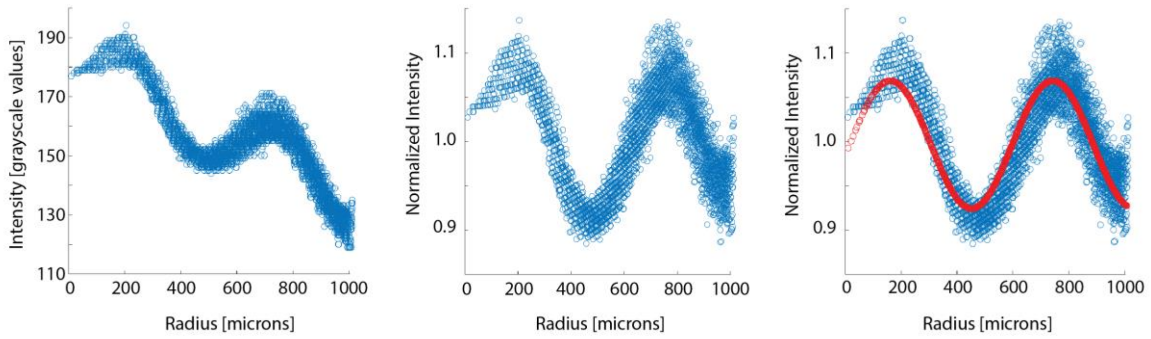

Supplementary Figure 9: Example of data normalization and curve fitting by RINGS.
A. The raw intensity values from the image of a single colony, demonstrating intensity loss at increasing radius. **B.** Intensity values after normalizing by a second order polynomial model. **C.** The model fit (red) can be seen overlaid on raw data (blue).

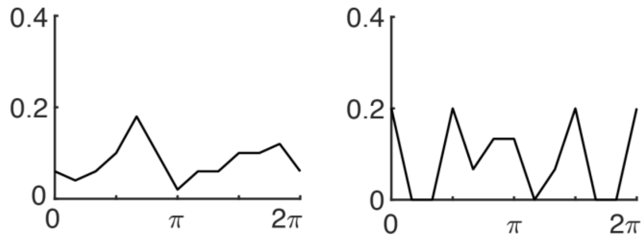

Supplementary Figure 10: Examples of datasets with angular deviation >1.2
Datasets with angular deviation >1.2 were routinely removed from downstream analyses of elapsed phase due to reduced confidence in the accuracy of circular mean calculations and the classification of colonies close to the circular mean ± π divide.

Supplementary Table 1: Strains used in this study.

**Strain**	**Source**	**Details**
LPT239	This study	*E. coli* MC4100 + pLPT234 + pLPT41
LPT320	This study	*E. coli* MC4100 + pLPT234 + pLPT145
LPT322	This study	*E. coli* MC4100 + pLPT234 + pLPT149
PAS715	This study	*E. coli* MG1655 DE(*lacI*) DE(*motA*) *rpsL* K42R + pLPT234 + pLPT145
PAS716	This study	*S.* Typhimurium LT2 + pLPT234 + pLPT145 (*streptomycin resistant through uncharacterized mutational selection).
PAS717	This study	*E. coli* Nissle 1917 DE(*lacI*) DE(*motA*) *rpsL* K42R + pLPT234 + pLPT145
PAS718	This study	*E. coli* MG1655 DE(*lacI*) DE(*motA*) *rpsL* K42R attTn7::pRNA1-mKate2 + pLPT234 + pLPT145

Supplementary Table 2: Peak-to-Trough ratio values. PTR as calculated from analysis of metagenomic samples sequenced by Illumina sequencing. Higher values correspond to higher estimated growth rates.

**Bacterial strain**	**mouse**	**Peak-to-Trough Ratio**			
		**Pre-admin**	**5.75h**	**16.25h**	**20.5h**
PAS715	1	<5% map	1.65	1.43	1.46
	3	<5% map	1.75	1.40	1.52
PAS716	1	<5% map	N/A	1.55	N/A
	2	<5% map	1.83	1.43	N/A
	3	<5% map	1.66	1.36	N/A

Supplementary Table 3: Plasmids used in this study

**Plasmid name**	**Source**	**Details**
pLPT234	This study	pSC101, Amp, repressilator 2.0 plasmid P_{LtetO1}-*cI*, P_R-*lacI*, P_{LlacO1}-*tetR*, P_{LtetO1}-*venus*, P_{LlacO1}-*cfp*, P_R-*mKate2*
pLPT41	[1]	ColE1, Kan, P_{tetO1} sponge plasmid
pLPT149	[1]	ColE1, Kan, P_{tetO1}+P_R sponge plasmid
pLPT145	[1]	ColE1, Kan, P_{tetO1}+P_R+P_{lacO1} sponge plasmid

Supplementary References

1. Potvin-Trottier L, Lord ND, Vinnicombe G, Paulsson J. Synchronous long-term oscillations in a synthetic gene circuit. *Nature* **538**, 514-517 (2016).
